# Implementing Topological Data Analysis for Monitoring Mass Transfer during Vacuum-Assisted Osmotic Dehydration of Apples

**DOI:** 10.1021/acsomega.5c00531

**Published:** 2025-07-08

**Authors:** Julio Emmanuel González-Pérez, Shengli Jiang, Oscar Jiménez-González, Víctor M. Zavala, Aarón Romo-Hernández, José Ángel Guerrero-Beltrán, Aurelio López-Malo, Nelly Ramírez-Corona

**Affiliations:** † Department of Chemical, Food and Environmental Engineering, 27806 Universidad de las Américas Puebla, Ex hacienda de Santa Catarina Mártir, San Andrés Cholula, Puebla, Mexico 72810; ‡ Tecnologico de Monterrey, School of Engineering and Sciences, Ave. Eugenio Garza Sada 2501 Sur, Col: Tecnológico, Monterrey, N.L. Mexico 64700; § Department of Chemical and Biological Engineering, 5228 University of Wisconsin-Madison, Madison, Wisconsin 53706, United States; ∥ Faculty of Gastronomy, Universidad Popular Autónoma del Estado de Puebla, 21 Sur No. 1103, Barrio de Santiago, Puebla 72410, Mexico

## Abstract

This study applies topological data analysis (TDA) to real-time experimental images of grape juice impregnation in apple samples with the goal of monitoring and quantifying the evolution of mass transfer under different processing conditions. We monitored the effective diffusivity (*D*) of water, solutes, and biocompounds during osmotic procedures using 40, 50, and 60 °Brix grape juice concentrate (GJC) by computing a topological descriptor of the real-time images known as the Euler characteristic curves (ECC). Osmotic procedures were osmodehydration (OD, absolute pressure, *P*_abs_ = 598 mmHg) and OD assisted with vacuum (ODVP, *P*_abs_ = 498 mmHg/10 min). The modified slope method for cubic geometry estimated *D* considering shrinkage and time evolution with *R* ^2^ > 0.80. Results show that samples treated with OD have higher shrinkage (20–25%) compared to those treated with ODVP. Water *D* was greater with ODVP and increased (*p* ≤ 0.05) at higher GJC concentrations. Solutes and total monomeric anthocyanins *D* were higher in OD and decreased with an increasing GJC concentration. Our TDA of real-time images revealed a double-peak structure of the ECC, which is characteristic of diffusion processes. We also found a simple topological metric (the difference between the maximum and minimum values of the ECC) that correlates positively with the diffusion coefficient *D* (*r* ^2^ > 0.7858) in OD experiments and negatively (*r* ^2^ < −0.9567) in ODVP at 60 °Brix. The TDA also indicated that the OD treatment at 40 °Brix resulted in uniform impregnation and redistribution of solutes within the impregnated samples after reaching osmotic equilibrium. The proposed TDA approach opens the door to nonintrusive, image-based monitoring of impregnation experiments, enabling high-throughput screening.

## Graphical abstract

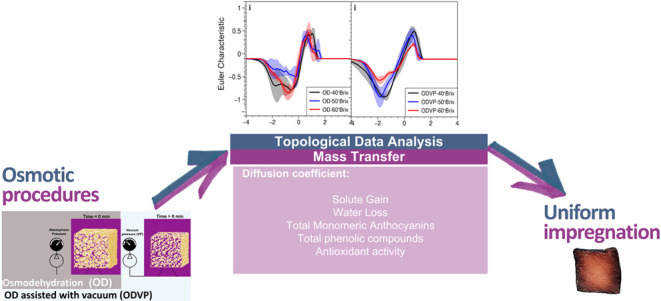

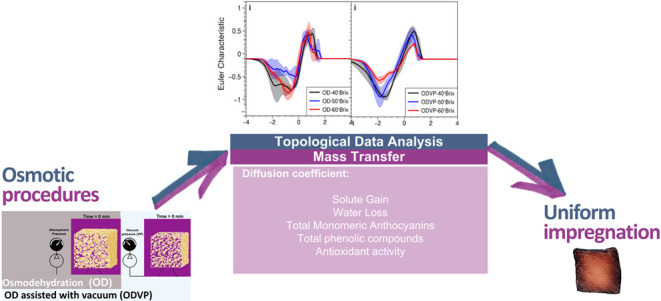

## 1 Introduction

Osmodehydration (OD) is a mass transfer process that allows simultaneous partial food dehydration and incorporation of solutes from an osmotic solution. This process differs from impregnation processes, since OD ends when the osmotic pressure of the sample and the osmotic agent reach equilibrium. A disadvantage of OD is that as the sample loses water, it tends to leach certain bioactive compounds from the impregnated matrix. To address this drawback, osmotic solutions rich in bioactive compounds have been used to compensate for the compounds leached during the process. A disadvantage of solute impregnation is that solids are deposited on the product surface, impeding water loss from the central part of dehydrated food. Furthermore, given that OD products tend to shrink more rapidly during the initial times, studies have recommended employing pretreatment with vacuum pulses to enhance solute incorporation and avoid morphometric changes. Vacuum pulse-assisted osmodehydration (ODVP) is a predrying method used to increase water loss compared with atmospheric pressure processes.^1^ ODVP studies have reported increases in the solute gain (SG) using isotonic solutions.^2^ This method also reduces crust formation during convection drying and increases water removal during the drying of impregnated fruits and vegetables.^3,4^ In addition, it has been reported that ODVP can lead to a reduction in water activity and consequently to an improvement in the quality of further dried or frozen products,^4^ reducing the pH value to increase the food stability by the impregnation with acid solutions,^5^ or reducing the oxygen presence to avoid browning of foods due to enzymatic and oxidative browning.^4^

Recent studies have assessed fruit juice concentrates as hypertonic solutions for OD of fruits and vegetables due to their high content of soluble solids and bioactive compounds. These compounds not only facilitate the dehydration process but also enable the incorporation of beneficial compounds into the final product. ODVP has also been implemented for celery roots enriched with bioactive compounds from onion, kale, and celery stalk juice,^3^ nonhydrolyzed and hydrolyzed tara gallotannins were vacuum-impregnated in potato chips to mitigate acrylamide formation.^6^ The process of vacuum impregnation of apples with aloe vera juice led to a reduction (lixiviation) in the content of bioactive compounds (vitamin C and total phenolic compounds).^1^ However, only a few studies have characterized the mass transfer rate in vacuum-assisted osmodehydration or described the evolution of impregnated bioactive compounds. The kinetics of osmosis processes demonstrate a rapid increase in mass transfer parameters until reaching a peak value under equilibrium conditions.^5^ Eyarkai Nambi et al.^7^ proposed first-order models to describe the degradation kinetics of bioactive compounds due to blanching of beetroots, green peas, eggplants, and green peppers at different temperatures (70–90 °C).

Morphological changes in the samples during processing impregnation of colored solution have been characterized by using real-time image analysis of apple tissue staining.^8,9^ Although most of these image analyses can aid in monitoring the impregnation grade, existing approaches provide limited information about visual changes in the sample. In this regard, Topological Data Analysis (TDA) provides a powerful tool for examining the morphological characteristics of images. TDA’s core idea is to quantify the shape of the images by quantifying connected and hole structures.^10^ TDA has proven to be effective in analyzing complex data sets in fields such as biology,^11^ and physics.^10^ A powerful TDA descriptor known as the Euler Characteristic curve (ECC) has been shown to be particularly effective at quantifying the shape/morphology of images.^12^ The ECC is an interpretable descriptor of topological spaces defined by data objects; ECC has been used to analyze structures of soft gelatin,^13^ characterization of the permeability of porous media,^14^ and the morphology of some crystals of carbohydrates (monosaccharides, disaccharides, and polysaccharides).^15^

This study aims to implement a new approach based on topological image analysis to evaluate apple tissue impregnation under different processing conditions. TDA extracts the ECC of real-time images (e.g., textures and bright/dark domains resulting from different levels of impregnation) and summarizes this information in the ECC. The ECC is a topological summary that is used to monitor the evolution of tissue staining due to osmotic dehydration and can help identify changes in the spatial distribution of the impregnated osmotic solution over time and as a function of the impregnation conditions. The obtained results were correlated with the dynamic behavior of effective diffusivity coefficients during osmotic dehydration with a vacuum pulse (ODVP) and OD of apple cubes with grape juice concentrate. Besides water and solute mass transfer, the antioxidant activity (AA), total phenolic compounds (TPC), and total monomeric anthocyanins were also monitored and described during the process. Our results indicate that a simple topological metric (the difference between the maximum and minimum peak values of the ECC) can be correlated to the diffusivity coefficients of water, solutes, and the bioactive components, thus paving the way to nonintrusive, image-based monitoring of impregnation experiments.

## 2 Materials and Methods

### 2.1 Raw Materials

Fresh Granny Smith apples (*Malus domestica* L.) were used as the food matrix. These were purchased from a local market in San Andrés Cholula, Puebla, Mexico. Fruits (0.84 ± 0.01 g water/g product, 13.1 ± 0.3 °Brix, and *a_w_* = 0.984 ± 0.001) were washed, sanitized (with 100 mg/L peracetic acid solution during 10 min), peeled, and cut into cubic pieces (approximately 1.2 cm × 1.2 cm × 1.2 cm). Grape juice concentrate (*Vitis vinifera*, cv. Victoria, 69.1 ± 1.0 °Brix) was obtained from the Casa Leal vineyard (Jesús María, Aguascalientes, Mexico). Osmotic solutions were prepared by diluting grape concentrate with distilled water at concentrations of 40, 50, and 60 °Brix.

### 2.2 Osmotic Processing

Osmotic processing of apple cubes involves osmodehydration (OD) under atmospheric pressure (598 mmHg) and vacuum-pressure-assisted osmotic dehydration (ODVP). The osmotic solution-to-food matrix ratio was maintained at 10:1 (w/w*)* to avoid significant dilution of the osmotic medium,^16^ and a temperature of 40 ± 0.5 °C. The osmotic pressure temperature was adjusted according to the temperature of the osmotic solution. It was measured using a digital K-type thermocouple thermometer (Thermocouple Meter, 20250-03, DIGI-SENSE, USA) and controlled with a water bath (Büchi, B-300 Base, Flawil, Switzerland). All experiments were conducted in triplicate.

The OD experiments were conducted individually with different immersion times ranging from 0 to 1440 min. A group of two apple cubes were completely immersed in the preheated osmotic solution (40 °C). OD was performed in cylindrical vessels, which was heated in a water bath (Büchi, B-300 Base, Flawil, Switzerland).

For ODVP, cylindrical glass test tubes (2 cm in diameter and 15 cm in height) with a food matrix immersed in an osmotic solution were subjected to a pressure reduction stage and an ambient pressure stage. In the first stage, the system pressure was reduced to absolute pressure of 498 mmHg for 10 min, followed by 5 min relaxation period at local atmospheric pressure (598 mmHg).^17^ Subsequently, osmodehydration continued until an osmotic equilibrium state was reached (no significant difference between the water activity of the sample and the osmotic agent).^5^ The pressure changes were controlled using a desiccator (15 cm in diameter and 27.5 cm in height) and a vacuum pump (Büchi, V-300, Flawil, Switzerland). The system temperature was maintained using a water bath (Büchi, B-300 Base, Flawil, Switzerland) containing 2.5 L of water, following the supplier’s usage recommendations.

After each immersion time, the samples were removed from the osmotic solution. The osmotic process was halted by immersing the samples in a cold distilled water bath (4 °C) for 10 s.^16^ Excess liquid from the samples was gently wiped from their surface using absorbent paper. Samples were weighed, and their moisture content before and after the osmotic process was used to calculate water loss and solute gain. In addition, the kinetics of water activity, total soluble solids, shrinkage characteristics, and bioactive compounds (antioxidant activity, total phenolic compounds, and total monomeric anthocyanins) were determined.

### 2.3 Water Activity and Total Soluble Solids

Water activity (*a_w_*) determinations were performed based on the dew point using an electronic hygrometer (Aqua Lab, 4TEV model, USA). The measurements were determined at a constant temperature (25 ± 0.5 °C). Total soluble solids of samples were measured with a digital refractometer (Atago Co., Pocket PAL-RI, Tokyo, Japan) and reported in °Brix.^18^

### 2.4 Shrinkage Characteristics

Shrinkage characteristics of apple cubes were analyzed with dimensionless volume (*V*/*V*_0_) according to eq 1. In eq 1, it is assumed that the cubic geometry remains undeformed throughout the process. The samples’ volume (*V*, m^3^) was determined using dimensions (*L*_1_, *L*_2_, and *L*_3_, m) of the cube. These dimensions were measured by using low-pressure digital Calipers (Truper, model CALDI-MP, Mexico).

1 $$\frac{V}{V_{0}}=\frac{L_{1}\times L_{2}\times L_{3}}{L_{1,0}\times L_{2,0}\times L_{3,0}}$$

where *L*_1_, *L*_2_, and *L*_3_ are the sample lengths (m) and *V* is the volume (m^3^) at a specific immersion time; *L*_1,0_, *L*_2,0_, and *L*_3,0_ are the initial lengths and *V*_0_ initial volume of the fresh cube.

### 2.5 Moisture Content

The moisture content of the samples (*Y*) was determined by oven drying (105 °C) until constant weight.^19^ Equations 2 and 3 were used to report on wet (*w.b.*) and dry (*d.b.*) basis, respectively.

2 $$M_{k}=\frac{g\;\mathrm{water}}{g\;\mathrm{fresh}\;\mathrm{sample}}={w.b.,}_{k=0\;\mathrm{or OP}}$$

3 $$\frac{w.b.}{1-w.b.}=d.b$$

### 2.6 Mass Transfer Properties

Mass transfer properties of the samples were determined considering negligible solid leaching out. Water loss (WL) and solute gain (SG) of samples during the osmotic process were calculated with eqs 4 and 5.^5^

4 $$\mathrm{WL}=\frac{m_{p0}M_{0}-m_{\mathrm{OP}}M_{\mathrm{OP}}}{m_{p0}}$$

5 $$\mathrm{SG}=\frac{m_{\mathrm{OP}}\left(1-M_{\mathrm{OP}}\right)-m_{p0}\left(1-M_{0}\right)}{m_{p0}}$$

where *m*_*p*0_ and *m*_OP_ are the weight (g) of fresh and osmotically treated samples at a certain immersion time, respectively, and *M*_0_ and *M*_OP_ are the mass fraction of moisture of fresh and osmotically treated samples at a certain immersion time on a wet basis, respectively.

### 2.7 Bioactive Compounds

#### 2.7.1 Bioactive Compounds Extraction

Extracts of the samples subjected to osmotic processing prior to the analyses of antioxidant activity (AA), total phenolic components (TPC), and total monomeric anthocyanins (TMA) were prepared following the method described by González-Pérez et al.^20^ with some modifications. Briefly, 2.50 ± 0.18 g of processed samples were homogenized using a mortar with 10 mL of 0.1% HCl in ethanol. After 12 h of macerating in the dark, the resulting mixture was centrifuged at 12,000 *g* at 10 °C for 10 min. All analyses of bioactive compounds were performed using three independent samples for each treatment condition, with triplicate measurements for all analytical determinations. The average of the analytical triplicates was then used for statistical analysis.

#### 2.7.2 Antioxidant Activity

The 2,2-diphenyl-1-picryl-hydrazyl (DPPH) assay was performed to determine the antioxidant activity (AA) of the sample’s extracts. The DPPH assay was performed according to the method described by Brand-Williams et al.,^21^ with some modifications. Prior to the colorimeter assay, sample extracts were diluted 1:3 (v/v) with ethanol (99.5%) to ensure that the absorbance values were on the scale. Then, 20 μL of the resulting mixture and 200 μL of a 0.1 mM ethanolic DPPH solution were added and mixed in a 96-well plate. After 30 min in the dark at room temperature (25 ± 1 °C), the absorbance was measured at 517 nm in a microplate spectrophotometer (Multiskan Sky Microplate spectrophotometer, Thermo Scientific, USA). Trolox (6-hydroxy-2,5,7,8 tetramethyl-chrome-2 carboxylic acid 97%) concentration, [*Trolox*] was selected under the condition of absorbance value ranging from 0 to 100 mg/L to plot a standard curve (*y* = 0.5944 × [*Trolox*] + 0.1659; *y* = 100 × (Abs_DPPH_ – Abs_sample_)/Abs_DPPH_; *R* ^2^ = 0.998). Finally, the results were expressed as mg of Trolox equivalents (TE)/100 g *d.b.* of product.

#### 2.7.3 Total Phenolic Compounds

The Folin-Ciocalteu method was used to determine the total phenolic compounds (TPC) of the sample extracts. The method was developed according to Singleton& Lamuela-Raventos,^22^ who adapted this for measuring in a 96-well microplate spectrophotometer (Multiskan Sky Microplate spectrophotometer, Thermo Scientific, USA). Sample extracts were diluted 1:6 (v/v) with distilled water before the method. 20 μL of the resulting mixture and 100 μL of Folin-Ciocalteu (0.1 M) were added to a 96-well plate and shaken for 3 s using a microplate spectrophotometer. After 3 min in the dark at room temperature, 100 μL of 2 M Na_2_CO_3_ was added to the mixture, and the mixture was shaken for 3 s. After 30 min, absorbance was measured at 765 nm. Different concentrations of gallic acid ([GAE]) were selected under the condition of absorbance values ranging from 0 to 50 mg/L to plot a standard curve (*y* = 0064 × [GAE] + 0.0008; *R* ^2^ = 0.980). Finally, the results were expressed as mg of gallic acid equivalents (GAE)/100 g *d.b.* of the product.

#### 2.7.4 Total Monomeric Anthocyanins

A pH-differential method was used to determine total monomeric anthocyanins (TMA) according to Giusti& Wrolstad^23^ with some modifications. Briefly, sample extracts were diluted 1:4 (v/v) with distilled water before the analysis. Diluted extracts were mixed with buffer solutions of pH 1 or 4.5 at a ratio of 1:4 (v/v). The absorbance of 200 μL of resulting mixtures was measured at 520 and 700 nm using a 96-well microplate spectrophotometer. TMA was calculated based on a malvidin-3,5-diglucoside molar extinction coefficient of 10,700 L/mol cm and a molecular weight of 655.4 g/mol.^24^ Results were expressed as mg malvidin-3,5-diglucoside/100 g *d.b*. of the sample.

### 2.8 Modeling of Mass Transfer Kinetics

The average experimental data were modeled with Matlab R2024a (Matlab Statistics Toolbox 7.3, MathWorks, Inc., Natick, MA, USA) using an analytical solution of the Fick second law, which estimates the water, solute, total soluble solids, and AA, TPC, and TMA effective diffusion. Experimental mass transfer data (*w*) of TSS, AA, TPC, and TMA were expressed as the mass change of the diffusing substance per mass of a fresh sample as described in eq 6. Equation 6 indicates the increase in mass transfer of the different analyzed *j* species (*Y*_*j*,OP_).

6 $$Y_{j,\mathrm{OP}}=w_{j,\mathrm{OP}}-w_{j,0}$$

where *w*_*j*,0_ and *w*_*j*,OP_ are the mass transfer data of fresh and osmotically treated samples at certain immersion time, respectively; subscript OP represents osmotic process type (OD or ODVP); and subscript *j* represents water loss, total soluble solids, AA, TPC, or TMA.

Equilibrium conditions of the increasing of different mass transfer parameters (*j*) were estimated using Peleg′s model^25^ described in eqs 7 and 8 by nonlinear regression:

7 $$Y_{j,\mathrm{OP}}=Y_{j,0}+\frac{t}{k_{1}+k_{2}t}$$

where *k*_1_ is the reciprocal of rate constant in s/(g water/g fresh product) for WL, s/(g solutes/g fresh product) for SG, s/(mg TE/100 g product *d.b*.) for AA, s/(mg GAE/100 g product *d.b*.) for TPC or s/(mg Mlv/100 g product *d.b*.) for TMA; *k*_2_ is constant of capacity in 1/(g water/g fresh product) for WL, 1/(g solutes/g fresh product) for SG, 1/(mg TE/100 g product *d.b*.) for AA, 1/(mg GAE/100 g product *d.b*.) for TPC or 1/(mg Mlv/100 g product *d.b*.) for TMA; *Y*_*j*,0_ is the initial condition of increasing different mass transfer parameters in g water/g fresh product for WL, g solutes/g fresh product for SG, mg TE/100 g product *d.b*. for AA, mg GAE/100 g product *d.b*. for TPC or mg Mlv/100 g product *d.b*. for TMA. The equilibrium condition in g water/g fresh product for WL, g solute/g fresh product for SG, mg TE/100 g product *d.b*. for AA, mg GAE/100 g product *d.b*. for TPC, or mg Mlv/100 g product *d.b*. for TMA was related to the Peleg coefficient constant *k*_2_ as follows:

8 $$Y_{j,e}=Y_{j,0}+\frac{1}{k_{2}}$$

Dynamic diffusion was calculated using the modified slope method for cubic geometry as described in eqs 9 and 10.^5^ This method proposes replacing the Fourier mass transfer number (τ_*j*_ = *D*_*j*_ *t*/*L*_0_) with a comprehensive definition that includes both diffusivity and variable characteristic length (τ_j_^*^) (eq 11). In this analysis, the materials considered were uniform and isotropic with water, solutes, antioxidant activity, total phenolic compounds, and total monomeric anthocyanins as diffusing substances. Additionally, the external resistance was deemed negligible. Finally, the weighted average diffusivity value for each substance was determined with eq 11:

9 $$\Psi_{j}=\frac{8^{3}}{\pi^{6}}\sum_{i=0}^{\infty }\frac{1}{{\left[2i+1\right]}^{6}}\exp\left[-\frac{{3\left[2i+1\right]}^{2}\pi^{2}}{4}\tau_{j}^{*}\right]$$

10 $$\Psi_{j}=1-\frac{Y_{j,\mathrm{OP}}}{Y_{j,e}}$$

$$\tau_{j}^{*}=\int_{0}^{t}\frac{D_{j}}{L^{2}}dt$$

with $L=L_{0}{\left(\frac{V}{V_{0}}\right)}^{1/3}$ , *L*_0_ = 0.00609 ± 0.00016 m, in m; *t*, time in s; *D*_*j*_, effective coefficient of *j*-substance as a function of the concentration of *j*-substance in m^2^/s; the substance *j* is water, solutes, antioxidant activity, total phenolic compounds, or total monomeric anthocyanins.

11 $$D_{j}=\frac{\int_{0}^{1}\int_{0}^{1}\int_{0}^{1}D_{j}d\Psi_{j}d\Psi_{j}d\Psi_{j}}{\int_{0}^{1}\int_{0}^{1}\int_{0}^{1}d\Psi_{j}d\Psi_{j}d\Psi_{j}}$$

The effect of neglecting shrinkage in the estimation of diffusion was analyzed by examining the relationship between diffusion without shrinkage (eq 12) and diffusion with shrinkage (eqs 9–11) during the process, referred to as *e*(*D*). The effective diffusion in a solid without shrinkage was determined using an analytical solution to the mass transfer described in eq 12. Through the linear regression of ln­(Ψ_*j*_) against time (*t*), the *slope* value was estimated. The effective diffusivity value was calculated with eq 13.

12 $$\ln\left(\Psi_{j}\right)=-\frac{3\pi^{2}D_{j}}{4\left(L_{0}^{2}\right)}t+\ln\left(\frac{8^{3}}{\pi^{6}}\right)$$

13 $$\mathrm{Slope}=-\frac{3\pi^{2}D_{j}}{4\left(L_{0}^{2}\right)}$$

### 2.9 Topological Image Analysis

TDA on the images was used to compare tissue impregnation during the osmotic dehydration of apples, both with and without a vacuum pulse. Digital images of 1 mm thick slices from the central part of an apple cube were captured at various immersion times using a camera positioned against a blue background alongside a reference object of known dimensions (Coolpix L810, Nikon Corp, Japan). A brightness in warm (3200 K) setting with a photographic studio (GLURIZ 64Leds, Hong Kong, China).^20^ The image analysis began with an image segmentation process to extract the sample from the raw image (Figure 1). Textural features of the image were obtained by applying a filtering process on a grayscale representation of the image, revealing connected and hole domains at varying light intensity levels. These topological features were summarized in Euler characteristic curves (ECCs).

**Figure 1:**
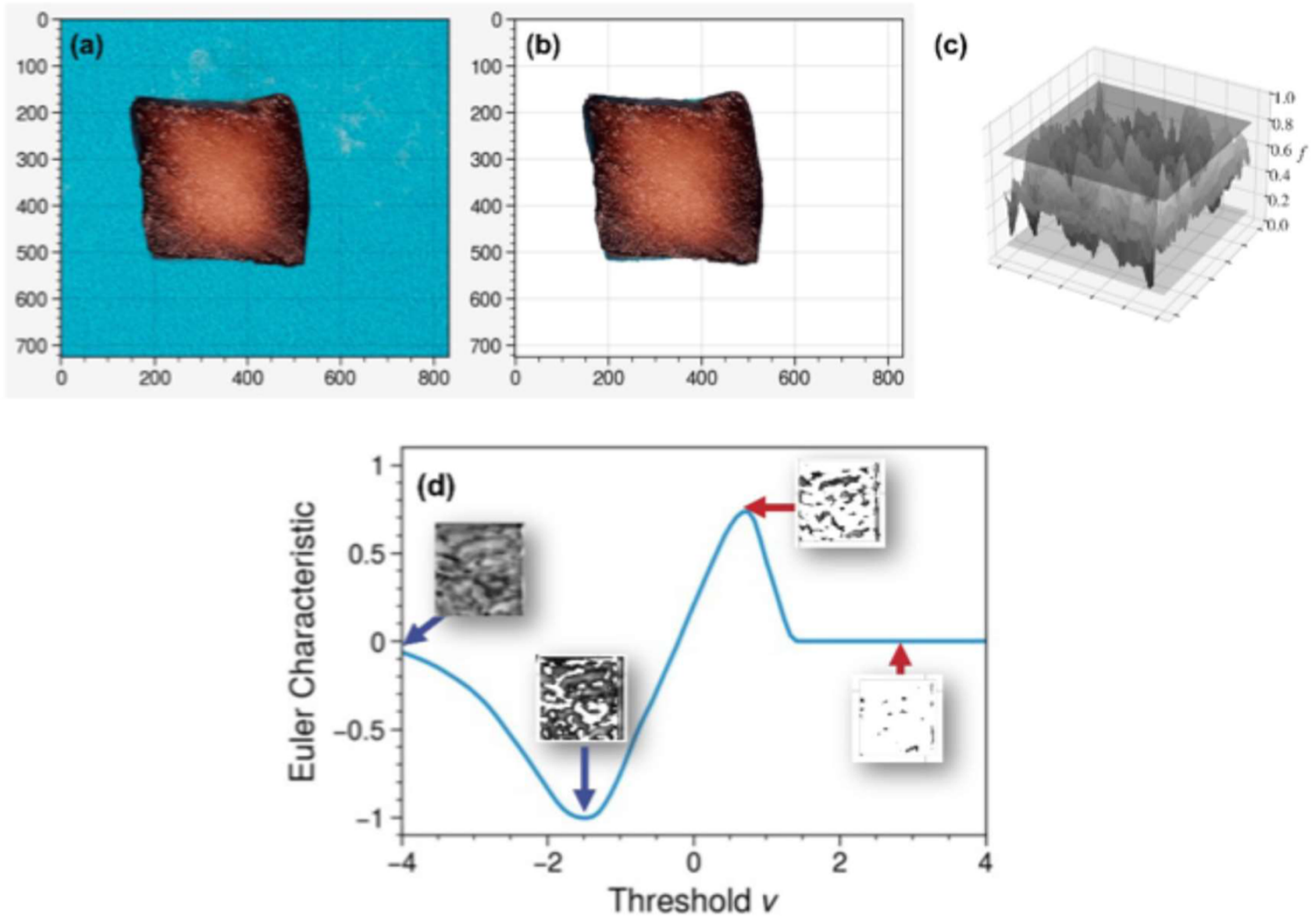
Image segmentation and topological data analysis for apples osmodehidrated with vacuum pulses in 40 °Brix of grape juice for 1440 min: (a) Original image, (b) segmented image without background, (c) field representation of the image, (d) filtering process of image, and topological summary (Euler characteristic curve) that captures the texture features of the image at different levels of light intensity.

The ECC provides a concise summary of the morphology of the apple samples as impregnation progresses.^12,26^ The ECC facilitates real-time monitoring and comparison under different impregnation conditions. Figure 1c illustrates the type of information contained in the ECC. Initially, the image is represented in 3D as a manifold or surface by adding light intensity as a third dimension. A filtering process is then applied to eliminate regions of the surface with a light intensity value below a certain threshold; this filtering process preserves connected domains of higher intensity and introduces holes. The ECC at a specific light intensity level is defined as the number of connected domains minus the number of holes.^26^ The holes correspond to darker regions of the image (more stained regions of the sample), while the connected components correspond to the brighter regions of the image (less stained regions of the sample). As such, the left part of the ECC in Figure 1d captures the number of dark regions or domains present in the sample and their size; specifically, the peak on the left side indicates the number of dark domains and their corresponding light intensity. The right peak indicates the number of bright domains and the corresponding light intensity. When the ECC has sharp peaks, it indicates that domains cover a narrow spectrum of light and that there are many domains; similarly, if the peaks are broad, it indicates that domains cover a broad spectrum of light and there are fewer domains. The double-peak structure of the ECC is characteristic of a Gaussian field, which is commonly found in diffusion processes. In other words, if the sample originates from a diffusive process, then the ECC will have a double-peak structure. The difference between the maximum and minimum peak values of the ECC was hypothesized to capture the effectiveness of diffusion during juice impregnation. This difference was used as a simple metric to examine the progression of tissue staining and was found to correlate strongly with the diffusivity coefficients for water, solutes, and bioactive components. This result is of significance, as it permits the nonintrusive monitoring (via images) of impregnation experiments.

### 2.10 Statistical Analysis

One-way ANOVA was conducted on the diffusivity coefficients obtained during osmotic procedures at various osmotic solution concentrations and between treatments (OD and ODVP). Additionally, one-way ANOVA was applied to the estimated increases in different mass transfer parameters gathered at various osmotic solution concentrations and between treatments (OD and ODVP). Means comparisons were made using the Tukey test (with 95% confidence). The kinetic model parameters were determined through nonlinear regression of the experimental data, utilizing the coefficient of determination (*R* ^2^) and Root Mean Squared Error (RMSE) to evaluate the fit of the function. The estimates of *k*_1_ and *k*_2_ were then used to calculate the *Y*_e_ term. Subsequently, *Y*_e_ was used as the basis for performing a one-way ANOVA to compare the groups. In all cases, the assumptions of the one-way ANOVA, including normality and uniformity of variance, were verified before performing the analysis. Finally, the relationship between the ECC, Max-Min, and the diffusivities of water, solutes, antioxidant activity, total phenolic compounds, and total monomeric anthocyanins were analyzed using Pearson’s correlation, considering 95% confidence. All statistical analyses were conducted with Minitab v.18 Statistical Software (Minitab, Inc., State College, PA, USA).

## 3 Results and Discussion

### 3.1 Osmodehydration Process

The equilibrium condition in mass transfer during osmotic procedures was analyzed by monitoring the evolution of water activity over time in the osmotic solution and apple cubes. The osmodehydration process was terminated at 810 min, at which no significant differences (*p* > 0.05) were observed between the water activity (*a_w_*) of the apple cubes and the osmotic solution, indicating that equilibrium was reached (Figure 2). The initial *a_w_* values of the osmotic solutions were 0.928 ± 0.002, 0.897 ± 0.005, and 0.858 ± 0.010 for 40, 50, and 60 °Brix, respectively. At the end of the osmodehydration process under atmospheric pressure, the *a*_*w*_ of the osmotic solution increased from 1 to 4% (*a*_*w OD40 °Brix*_ = 0.931 ± 0.005, *a*_*w OD50 °Brix*_ = 0.897 ± 0.006, *a*_*w OD60 °Brix*_ = 0.858 ± 0.009 para 40, 50, and 60 °Brix), this indicates that the osmotic solution was almost negligibly diluted by the water lost during the dehydration of the samples.^5^ Regarding the *a*_*w*_ of the osmotic solution from the vacuum experiments, no significant differences (*p* > 0.05) were shown with respect to the initial values. de Jesus Junqueira et al.^16^ found no significant differences between the *a*_*w*_ of slices (2 cm × 2 cm × 0.4 cm) of eggplant (*a*_*w*_ = 0.991) and a solution of 40 kg sucrose and 10 kg sodium chloride/100 kg (*a*_*w*_ = 0.836) after the vacuum pulse-assisted osmodehydration process (10 min at 160 mmHg and 290 min at atmospheric pressure, 759 mmHg). Lower *a*_*w*_ values in samples processed with vacuum pulse-assisted osmodehydration than with atmospheric pressure have been reported in the literature.^27^

**Figure 2:**
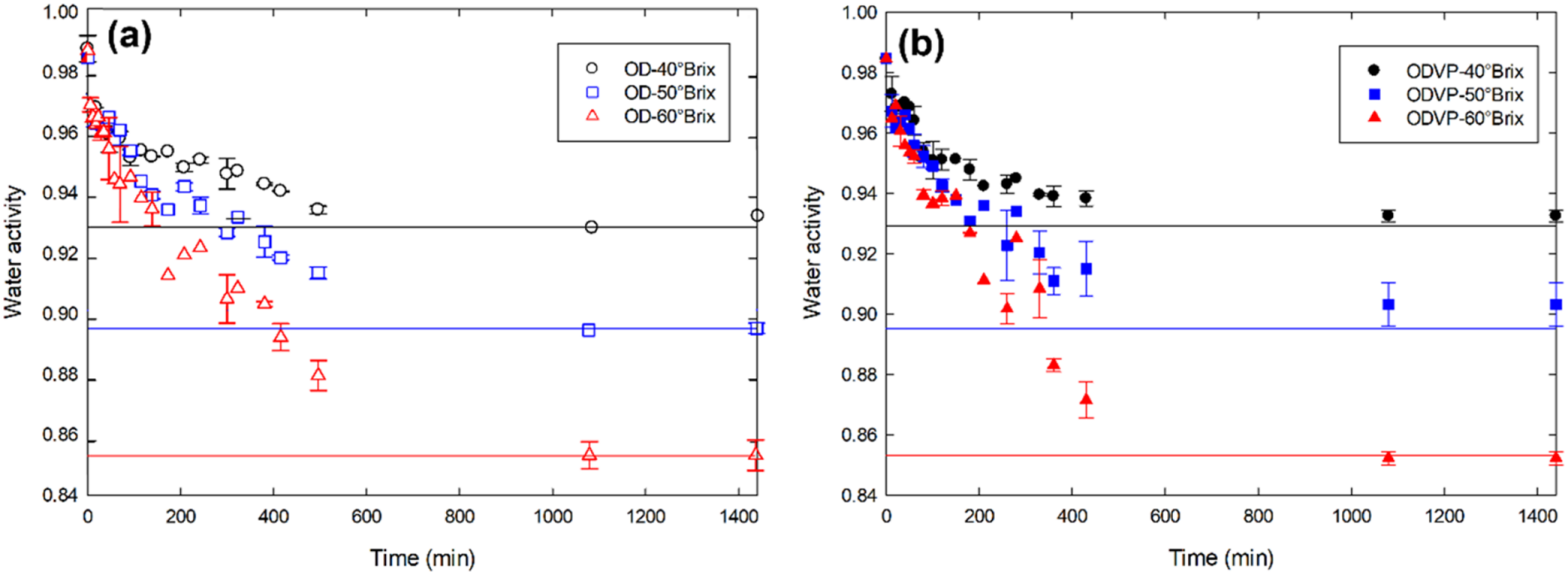
Evolution of water activity during osmodehydration (a) without (OD) and (b) with vacuum pulses (ODVP) at 40 °C. Dots and continuous lines correspond to the water activity of the sample and of the osmotic solution, respectively.

The evolution of the incorporation of total soluble solids during the osmotic procedures is shown in Figure 3a. All samples subjected to osmotic treatments exhibited values exceeding the initial value of 13.1 ± 0.3 °Brix. The values of total soluble solids increased significantly (*p* ≤ 0.05) with increasing osmotic solution concentration. On the other hand, it was observed that the incorporation of total soluble solids increased with the application of vacuum pulses (498 mmHg), especially during the first minutes of processing. According to González-Pérez et al.,^5^ the incorporation of solutes is important in osmotic dehydration processes because it prevents the collapse of the osmotic dehydrated sample, reducing the deformation and shrinkage of the product.

**Figure 3:**
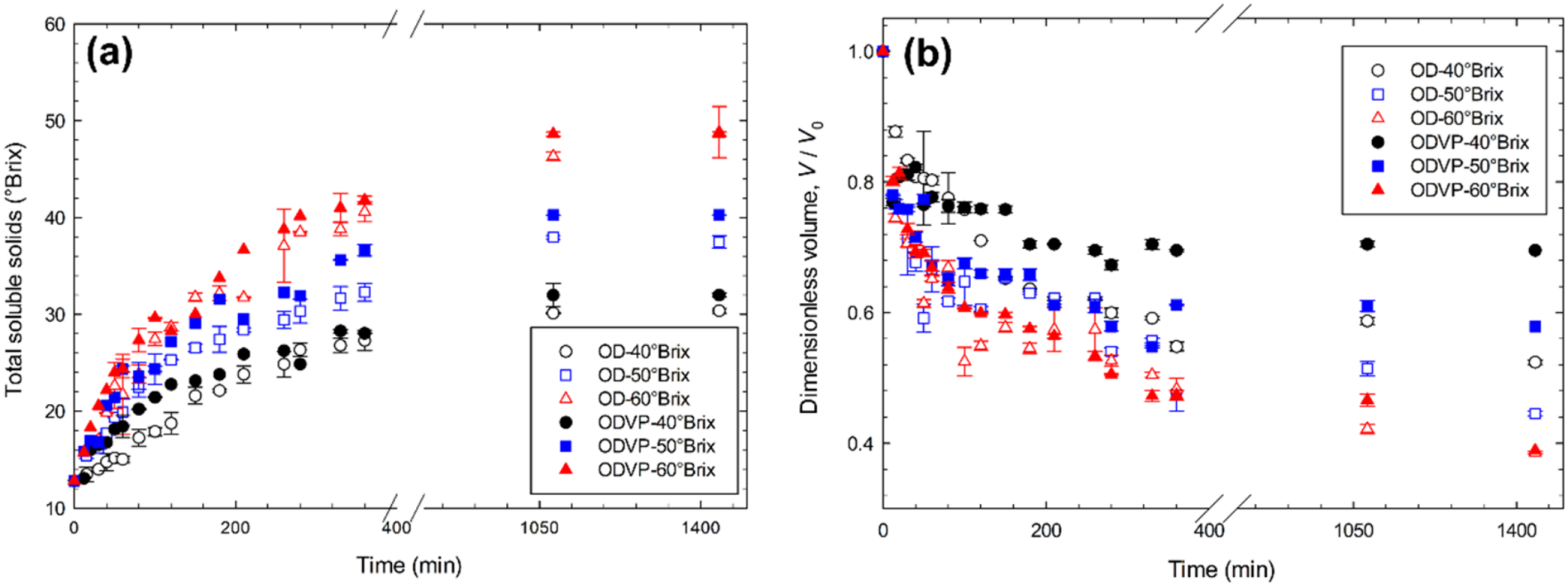
Evolution of (a) total soluble solids and (b) dimensionless volume during ODVP and OD of apple with grape juice concentrate.

In order to analyze the physical behavior of the samples during treatment, structural shrinkage was assessed by evaluating the dimensionless volume of apple cubes throughout the osmodehydration process (Figure 3b). The samples that shrank the most (*p* ≤ 0.05) were those treated with more concentrated solutions. Corrêa et al.^27^ associated the shrinkage of osmotically dehydrated samples with vacuum pulses to the water removed during the process. When comparing similar concentrations of osmotic solutions, it was observed that samples subjected to osmotic dehydration with vacuum pulses (25–60%) shrunk less than those treated at atmospheric pressure (45–60%), this phenomenon can be attributed to the fact that processes with vacuum pulses replace air and water with solutes faster, reducing the collapse of the product structure.^20^

During osmotic dehydration and vacuum-assisted osmodehydration, all samples were deformed, in comparison with the initial samples. Figure 4 shows that the deformation is due to a slight corner effect. In this deformation type, the center of each side shrinks more than the edges of the sample.^5,28^ No uniform impregnation is responsible for this type of deformation, as a high concentration of solutes on the external part of the samples reduces the water loss. In osmotically dried samples, this deformation appeared after 330 min for samples dried at 50 and 60 °Brix for grape juice. At 40 °Brix of grape juice, samples using vacuum-assisted osmotic dehydration showed this deformation at 810 min at 50 and 60 °Brix. González-Pérez et al.^5^ found that the apple osmodehydrated with 60 °Brix of concentrated apple juice showed corner effect deformation due to the concentration of solutes on the surface of the sample, which incorporation was slower than the elimination of water due to the high viscosity of the juice.

**Figure 4:**
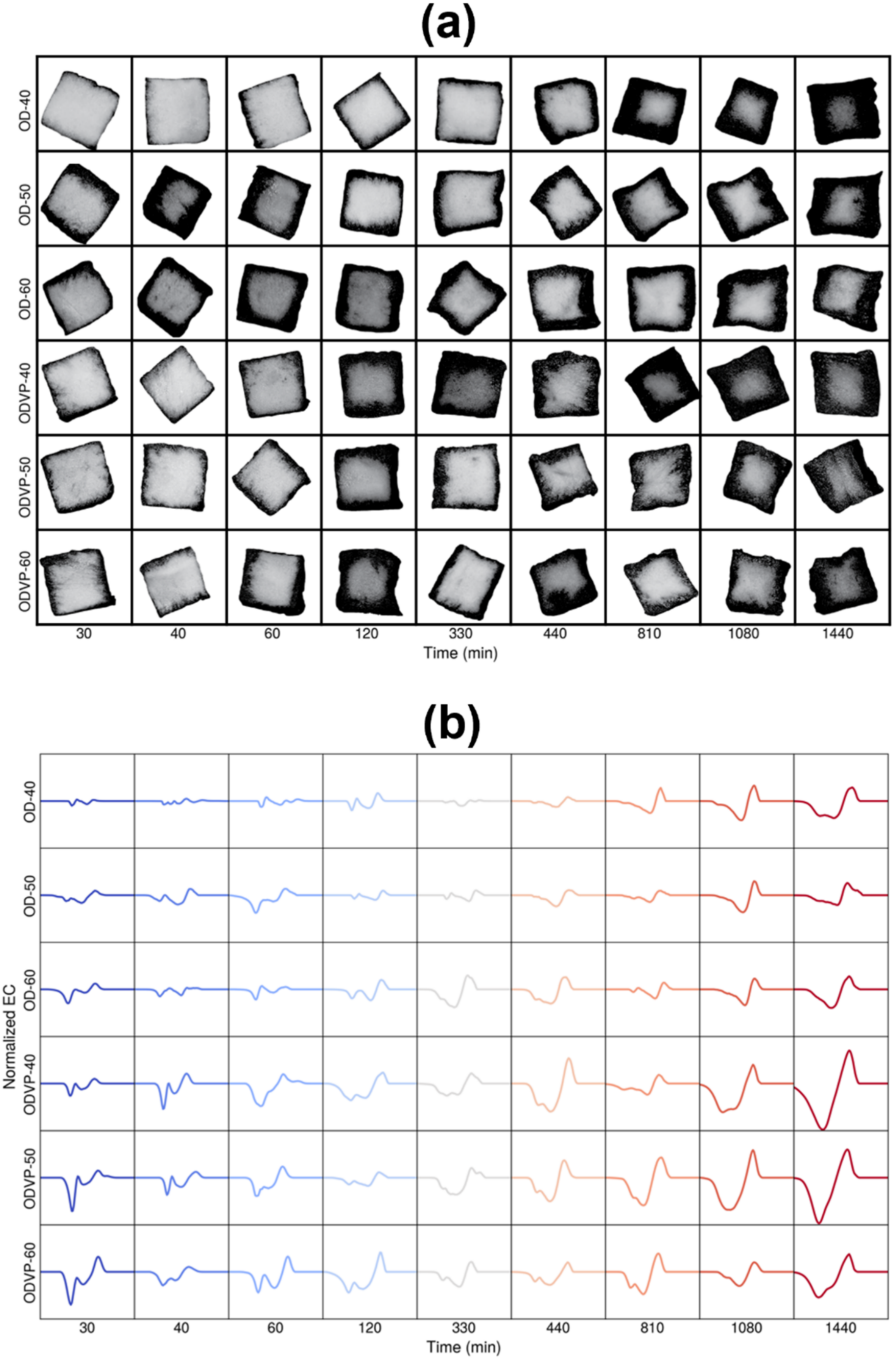
Temporal evolution of the image samples under different processing conditions. (a) Grayscale images and corresponding (b) topology evolution of image samples.

### 3.2 Image Topology Analysis of Tissue Impregnation and Mass Transfer Characteristics

The implementation of vacuum pulses before the osmosis process increased WL and increased with the rise in osmotic agent concentration (Table 1 and Figure 5). At 30 min of processing, samples subjected to ODVP exhibited higher SG values compared to those processed with OD. However, SG values did not demonstrate significant differences (*p* > 0.05) under equilibrium conditions between samples treated with vacuum pulses and those treated under atmospheric pressure. Corrêa et al.^27^ reported that ODVP removed 9–11% more water from Persimmon fruit processed with 30 and 45 °Brix fructose solutions compared to OD. Similarly, they found no significant differences in the values of SG when using a pretreatment with a vacuum. On the other hand, Moreno et al.,^29^ at 30 min of osmosis process, the use of vacuum pressure (37.5 mmHg for 5 min) increases SG compared to atmospheric pressure osmodehydration in apple (cv. Granny Smith) and pear (cv. Packham’s Triumph) cubes with 65 °Brix sucrose.^29^ Likewise, Junqueira et al.^30^ found that the use of vacuum pulses (absolute pressure 460.1 and 160.1 mmHg for 10 min at 35 °C) as pretreatment during osmodehydration of eggplant and beetroot in a solution of 40% sucrose and 10% sodium chloride (*a*_*w*_ = 0.836), reduced the rate of SG compared to samples processed at atmospheric pressure (absolute pressure 760.1 mmHg). In addition, at lower pressures, a reduction in the SG rate was observed. Finally, regardless of the process pressure, no significant differences (*p* < 0.05) were reported between the SG values reached at steady state during the osmotic procedures of persimmon fruit with fructose solutions^27^ or carrot, eggplant, and beetroot with 40% sucrose and 10% sodium chloride.^30^

**Figure 5:**
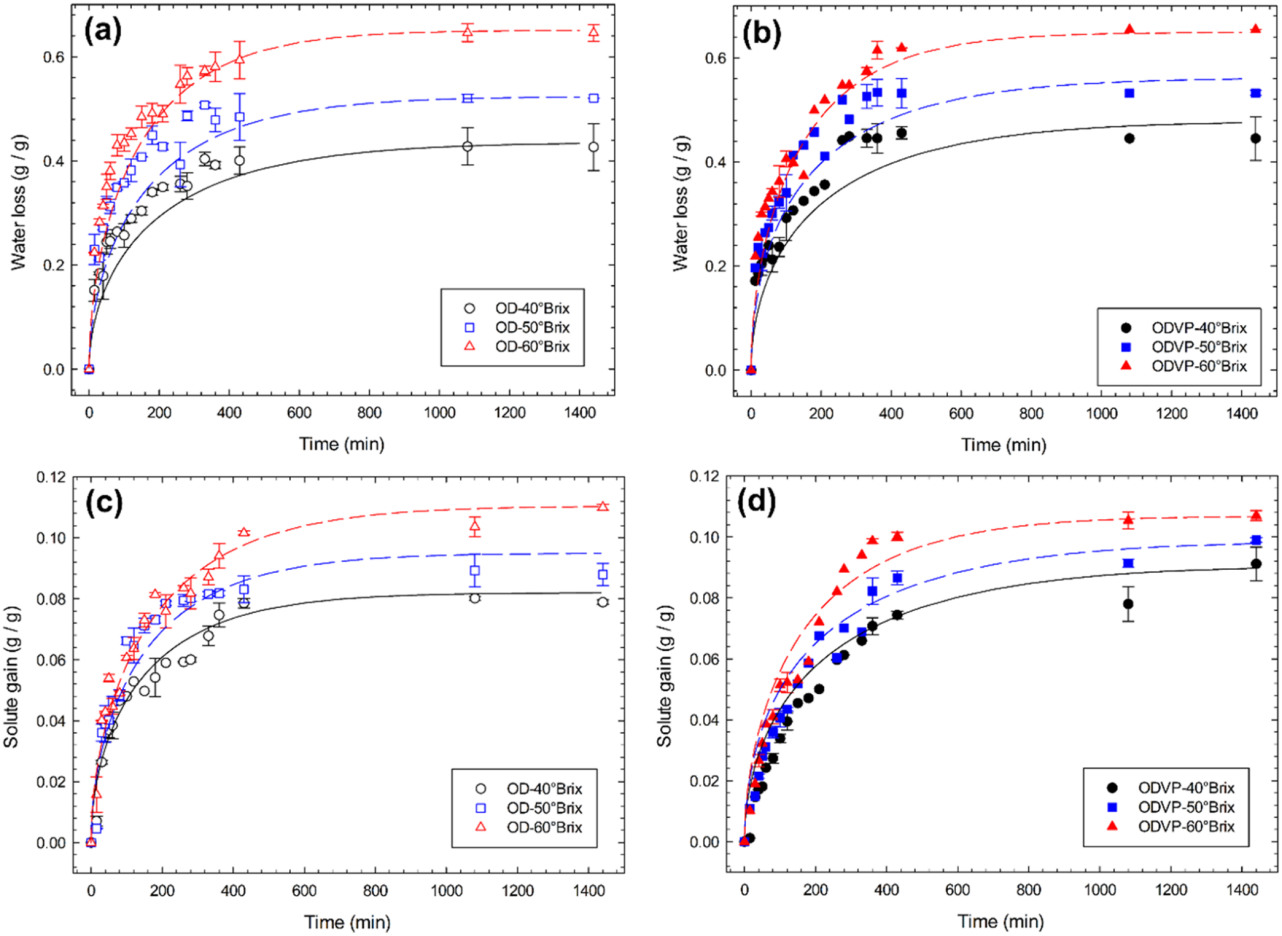
Evolution of mass transfer characteristics of water loss during (a) OD and (b) ODVP, and of solute gain during (c) OD and (d) ODVP of apple with grape juice concentrate at 40 °C.

**Table 1:** Increase of Different Mass Transfer Parameters at Equilibrium for Processed Apple by Osmodehydration (OD) and Osmodehydration Assisted by Vacuum (ODVP) at Different Concentrations (C)

			Increasing of different mass transfer parameters (*Y_j_*–*Y*_*j*0_)^a^									
Process	C (°Brix)	Peleg parameter	WL −WL_0_	Units	SG–SG_0_	Units	AA–AA_0_	Units	TPC–TPC_0_	Units	TMA–TMA_0_	Units
OD	40	*Y*_e_ = *Y*_e_*–*Y*_0_	0.436 ± 0.015cB	g water/g fresh product	0.082 ± 0.003cA	g solutes/g fresh product	129.89 ± 1.69bA	mg TE/100 g	190.18 ± 3.61cB	mg GAE/100 g	62.89 ± 3.97cA	mg Mlv/100 g
		*k*_1_	6753.13 ± 1007.38	s/(g water/g fresh product)	51810.00 ± 5172.13	s/(g solutes/g fresh product)	104.16 ± 55.81	s/(mg TE/100 g)	14.17 ± 0.33	s/(mg GAE/100 g)	17.39 ± 6.97	s/(mg Mlv/100 g)
		*k*_2_	2.29 ± 0.08	1/(g water/g fresh product)	12.15 ± 0.37	1/(g solutes/g fresh product)	0.008 ± 0.000	1/(mg TE/100 g)	0.005 ± 0.00	1/(mg GAE/100 g)	0.016 ± 0.001	1/(mg Mlv/100 g)
		*R* ^2^	0.972		0.988		0.946		0.964		0.979	
		RMSE	0.004		0.001		3.394		1.469		1.054	
	50	*Y*_e_ = *Y*_e_*–*Y*_0_	0.524 ± 0.022bF	g water/g fresh product	0.095 ± 0.003bE	g solutes/g fresh product	130.34 ± 0.07bE	mg TE/100 g	258.35 ± 4.05bF	mg GAE/100 g	79.07 ± 1.69bE	mg Mlv/100 g
		*k*_1_	4232.97 ± 789.29	s/(g water/g fresh product)	37190.00 ± 3643.24	s/(g solutes/g fresh product)	33.78 ± 16.06	s/(mg TE/100 g)	6.2465 ± 4.6931	s/(mg GAE/100 g)	15.18 ± 9.29	s/(mg Mlv/100 g)
		*k*_2_	1.91 ± 0.08	1/(g water/g fresh product)	10.56 ± 0.29	1/(g solutes/g fresh product)	0.007 ± 0.000	1/(mg TE/100 g)	0.0039 ± 0.0006	1/(mg GAE/100 g)	0.013 ± 0.001	1/(mg Mlv/100 g)
		*R* ^2^	0.959		0.984		0.954		0.887		0.955	
		RMSE	0.005		0.001		2.916		8.673		0.233	
	60	*Y*_e_ = *Y*_e_*–*Y*_0_	0.651 ± 0.017aI	g water/g fresh product	0.105 ± 0.002aI	g solutes/g fresh product	163.25 ± 2.66aJ	mg TE/100 g	606.87 ± 3.69aI	mg GAE/100 g	96.79 ± 0.12aI	mg Mlv/100 g
		*k*_1_	4057.13 ± 475.21	s/(g water/g fresh product)	37880.00 ± 3396.19	s/(g solutes/g fresh product)	18.13 ± 8.74	s/(mg TE/100 g)	4.55 ± 2.91	s/(mg GAE/100 g)	14.58 ± 0.00	s/(mg Mlv/100 g)
		*k*_2_	1.54 ± 0.04	1/(g water/g fresh product)	9.51 ± 0.25	1/(g solutes/g fresh product)	0.006 ± 0.000	1/(mg TE/100 g)	0.002 ± 0.00	1/(mg GAE/100 g)	0.010 ± 0.001	1/(mg Mlv/100 g)
		*R* ^2^	0.983		0.985		0.940		0.895		0.876	
		RMSE	0.004		0.001		3.281		18.025		2.687	
ODVP	40	*Y*_e_ = *Y*_e_*–*Y*_0_	0.479 ± 0.023hA	g water/g fresh product	0.093 ± 0.003hA	g solutes/g fresh product	45.87 ± 0.001hB	mg TE/100 g	282.86 ± 1.63hA	mg GAE/100 g	35.60 ± 0.12hB	mg Mlv/100 g
		*k*_1_	6434.47 ± 1030.57	s/(g water/g fresh product)	48500.00 ± 8912.91	s/(g solutes/g fresh product)	68.43 ± 37.55	s/(mg TE/100 g)	6.7208 ± 1.4264	s/(mg GAE/100 g)	45.07 ± 0.11	s/(mg Mlv/100 g)
		*k*_2_	2.09 ± 0.10	1/(g water/g fresh product)	10.80 ± 0.68	1/(g solutes/g fresh product)	0.021 ± 0.000	1/(mg TE/100 g)	0.0035 ± 0.0002	1/(mg GAE/100 g)	0.028 ± 0.001	1/(mg Mlv/100 g)
		*R* ^2^	0.962		0.959		0.935		0.824		0.923	
		RMSE	0.005		0.001		1.176		2.729		0.507	
	50	*Y*_e_ = *Y*_e_*–*Y*_0_	0.562 ± 0.022gE	g water/g fresh product	0.099 ± 0.002gE	g solutes/g fresh product	92.21 ± 0.00gF	mg TE/100 g	497.04 ± 1.68gE	mg GAE/100 g	49.59 ± 0.24gF	mg Mlv/100 g
		*k*_1_	4730.40 ± 597.23	s/(g water/g fresh product)	36160.00 ± 4147.68	s/(g solutes/g fresh product)	50.98 ± 20.84	s/(mg TE/100 g)	13.9883 ± 6.9522	s/(mg GAE/100 g)	31.82 ± 10.24	s/(mg Mlv/100 g)
		*k*_2_	1.78 ± 0.07	1/(g water/g fresh product)	10.15 ± 0.36	1/(g solutes/g fresh product)	0.011 ± 0.000	1/(mg TE/100 g)	0.0020 ± 0.0004	1/(mg GAE/100 g)	0.020 ± 0.001	1/(mg Mlv/100 g)
		*R* ^2^	0.970		0.978		0.953		0.894		0.898	
		RMSE	0.005		0.001		1.776		11.751		0.711	
	60	*Y*_e_ = *Y*_e_*–*Y*_0_	0.649 ± 0.001fJ	g water/g fresh product	0.107 ± 0.003fI	g solutes/g fresh product	191.60 ± 0.00fI	mg TE/100 g	582.65 ± 1.07fJ	mg GAE/100 g	61.72 ± 0.38fJ	mg Mlv/100 g
		*k*_1_	4522.84 ± 0.15	s/(g water/g fresh product)	28910.00 ± 2862.86	s/(g solutes/g fresh product)	7.76 ± 3.08	s/(mg TE/100 g)	11.9863 ± 5.7518	s/(mg GAE/100 g)	44.13 ± 11.27	s/(mg Mlv/100 g)
		*k*_2_	1.54 ± 0.00	1/(g water/g fresh product)	9.35 ± 0.27	1/(g solutes/g fresh product)	0.005 ± 0.000	1/(mg TE/100 g)	0.0017 ± 0.0003	1/(mg GAE/100 g)	0.016 ± 0.001	1/(mg Mlv/100 g)
		*R* ^2^	0.962		0.982		0.956		0.930		0.891	
		RMSE	0.006		0.001		1.971		133.302		0.733	

a Estimations are presented as coefficient ± standard errors. These standard errors indicate 95% of precision of estimated coefficients. *Y*_e_* is real mass transfer parameters at equilibrium condition; *Y*_e_ is the increasing of different mass transfer parameters at equilibrium; *k*_1_ and *k*_2_ are constant of rate and capacity, respectively; WL = water loss (g water/g fresh product); WL_0_ = 0 g water/g fresh product; SG = solid gain (g solutes/g fresh product); SG_0_ = 0 g solutes/g fresh product; AA = antioxidant activity (mg TE/100 g); AA_0_ = 550 ± 0.24 mg TE/100 g d.b.; TPC = total phenolic compounds (mg GAE/100 g); TPC_0_ = 415.97 ± 0.99 mg GAE/100 g d.b.; TMA, Total monomeric anthocyanins (mg Mlv/100 g); TMA0 = 0 mg Mlv/100 g. d.b. In the same column, lowercase letters (a, b, c, or f, g, h) indicate a significant difference (*p* ≤ 0.05) when comparing osmotic solution concentration at same treatment. Additionally, capital letters (A, B or E, F or I, J) indicate significant differences (*p* ≤ 0.05) when comparing the type of treatment at same osmotic solution concentration.

Evaluation of the internal morphology of apple samples subjected to different osmotic treatments revealed complex dynamics. It is reflected in the topological variations observed throughout the treatment. Figure 4a,b shows the corresponding grayscale images and ECC summarizing the topology of the images from the central part of the apple samples subjected to different osmotic treatments. In Figure S2, the evolution of the ECC is visualized and superimposed. This reveals that the evaluation of the sample morphology is complex, and thus dynamic differences across samples are difficult to interpret by pure visual inspection.

A comparison between ODVP and OD samples reveals that using vacuum leads to a more abrupt impregnation, as evidenced by ECC with sharper left and right peaks, indicating that impregnation is less uniform. This might result from more total solids accumulating at the boundary of the ODVP samples. Moreover, the topology of ODVP samples changes more abruptly than that of OD ones, suggesting that impregnation and deformation are stronger. It is also observed that the ECC of the ODVP samples is shifted slightly to the left (compared to the ECC of the OD samples); this suggests that domains are, in general, darker. Finally, the ECC for all samples exhibits a characteristic double-peak curve, which is a clear indication of the diffusive behavior. This is important because diffusive behavior is not readily apparent at the onset of impregnation.

During the osmotic procedures, the topological evolution of the apple samples revealed important differences in the impregnation dynamics, depending on the concentration of the solution used. In particular, the impregnation of OD-40 samples exhibits a significant acceleration at approximately 440 min and converges to a uniform dark texture (Figure 4), indicating efficient impregnation. This phenomenon can be attributed to the mass transfer curves of OD-40 demonstrating a constant velocity until the equilibrium state is achieved (Figure 3a). A similar dynamic evolution of topology is observed for the OD-50 and OD-60 samples, but the impregnation of the OD-60 sample occurs faster, resulting in a final state that is less uniform than that of OD-40. The dark domains located at the boundary of the ODVP samples exhibit accelerated development, which is reflected in the ECC by the presence of more prominent peaks that increase over time. In contrast, the peaks in the OD samples are less pronounced and occur at times close to the equilibrium state. Consequently, pronounced peaks of the ECC at the beginning of the ODVP process indicate a reduction in solute transfer and nonuniform impregnation, resulting in an oversaturation of solutes. Overall, all samples exhibit the same characteristic time scale, ranging from 330 to 440 min, during which the topology changes rapidly. González-Pérez et al.^20^ analyzed the apple tissue areas impregnated with grape juice during vacuum impregnation. Their image analyses utilized pixel intensity values, where 255 represented the highest intensity (light) and 0 represented the lowest intensity (dark). We found that pixel intensity values ranging from 175 to 0 indicated impregnated tissue as these values differed from those of the fresh sample, which ranged from 255 to 175. However, visual limitations led to conclusions suggesting that the product exhibited uniform impregnation, even when some items displayed darker pixel intensities on their outer portions. In the new proposed approach, the ECC enables us to identify when a reduction in solute transfer and nonuniform impregnation results in solute oversaturation.

Although the apple cube had achieved mass transfer equilibrium (Figures S1 and S2), all the ECC showed variations at process times >810 min (Figures 3 and S2). This could be attributed to the internal diffusive processes of certain solutes and water in the internal part of the apple cube, indicating a grouping of the internal water and solute composition of the product. These variations were not possible to measure with the mass transfer parameters of the product due to no significant differences being reached (*p* > 0.05). Guz et al.^9^ attribute a reduction of liquid diffusion in the pores of apple tissue due to the viscosity of the osmotic solution, which can explain why the ECC showed differences after reaching the equilibrium condition. González-Pérez& López-Malo^31^ found that the rate of water removal during convective drying of apples impregnated with grape juice under atmospheric conditions was reduced due to the formation of a crust on the surface of the product, which prevented the removal of the water present in the center of the product. Therefore, if vacuum-impregnation with viscous osmotic solutions is to be applied as a drying pretreatment, analysis of the ECC after osmotic equilibrium conditions are reached is recommended to reduce the water concentration in the center of the impregnated sample and increase water removal during the convective drying process.

The analysis of uniform impregnation during osmotic treatment was carried out by evaluating the differences between the maximum and minimum values of the ECC peak (Figure 6). Small values of these differences indicate more uniform impregnation, while higher values indicate a more nonuniform impregnation. At 30 min of treatment, the samples subjected to ODVP (0.291–0.658) showed higher differences than OD (0.144–0.182), indicating that applying vacuum as a pretreatment to OD allows incorporation of juice in a larger area. Subsequently, intermittent increases and decreases occur before 200 min. This behavior could be because the viscosity of the juice is higher than that of the apple water, so water loss was faster during that time interval than solute gain.^32,33^ After 400 min and at concentrations of 40 and 50 °Brix, regardless of the treatment, it was observed that the difference tended to increase, indicating an increase in juice incorporation. In the OD experiments, a reduction in the ECC Max-Min difference was found, possibly due to the redistribution of solutes within the samples. In the case of OD samples with 60 °Brix, the previously described behavior is maintained, but the increase in the difference was observed from 800 min; the process is slower because the viscosity is higher at higher concentrations. The ODVP experiments at 60 °Brix show more evident fluctuations, which would indicate that a cluster of osmotic agents forms on the outside of the product, slowly diffuses toward the center of the product, and then another cluster of osmotic agents forms again on the surface of the product. It is important to emphasize that the ECC trends shown in Figure 6 do not reveal any dynamic trend of the morphology of the samples; however, as we will describe shortly, the ECC values are strongly correlated to diffusion coefficient.

**Figure 6:**
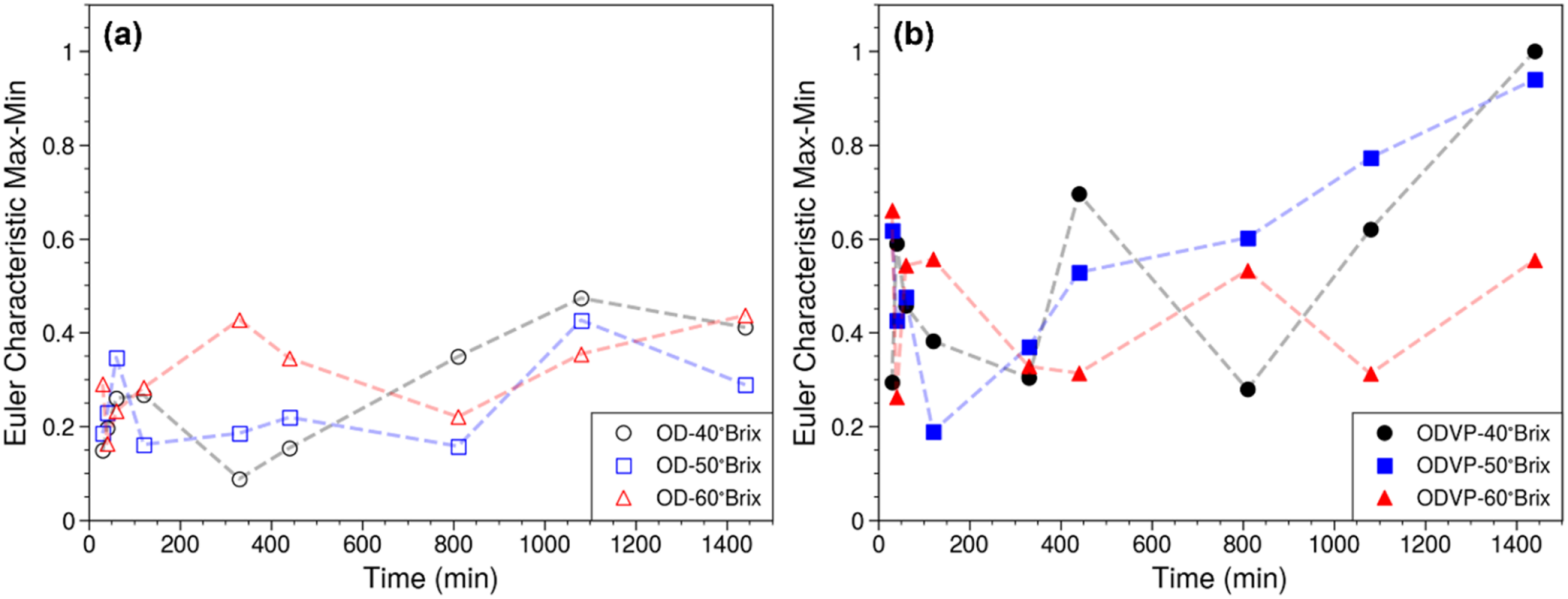
Temporal evolution of normalized Euler characteristic curve peak differences during (a) (OD) and (b) ODVP.

Bioactive compound values of fresh apple var. Granny Smith were 448 ± 0.24 mg TE/100 g *d.b*. and 415.97 ± 0.99 mg GAE/100 g *d.b*. The bioactive compound values in the grape juice were 179.37 ± 4.04 mg Malvidin-3,5-diglucoside/100 g product *d.b*., 734.88 ± 0.86 mg TE/100 g product *d.b*., and 1388.49 ± 10.70 mg GAE/100 g product *d.b*. Table 1 and Figure 7 show the equilibrium values (*Y*_e_) and the kinetics of incorporation of bioactive compounds of apples subjected to osmosis processes. The application of 30 min of vacuum pressure prior to the osmodehydration process increased the incorporation of bioactive compounds compared with samples processed without pretreatment. In addition, samples osmodehydrated at ODVP with 60 °Brix solutions had an increase in the AA and total phenolic compounds and a reduction in the total monomeric anthocyanins compared to those treated at atmospheric pressure. This reduction could be attributed to the viscosity of the osmotic solutions. It impedes the incorporation of total monomeric anthocyanins. González-Pérez et al.^20^ reported that vacuum impregnation of total monomeric anthocyanins in apples decreased due to solute accumulation on the product’s external surface, thereby reducing the incorporation of total soluble solids and total monomeric anthocyanins. On the other hand, Liu et al.^34^ identified that anthocyanin monomers, including delphinidin, cyanidin, petunidin, peonidin, and malvidin from blueberry powder, predominantly degraded between 41 and 48.5 °C. Additionally, several studies have demonstrated that elevated temperatures expedite the degradation of anthocyanins in grapes^35^ and lychees.^36^ It has been reported that the total monomeric anthocyanins content in grape juice decreases by 25.9 to 36.9% following 30 min of apple osmodehydration at 40 °C.^37^ Elevated temperatures tend to disrupt certain functional group bonds involved in methoxylation, glycosylation and acylation,^36^ or accelerate enzymatic reactions due to polyphenol oxidase, peroxidase or anthocyanin-β-glycosidase.^38,39^

**Figure 7:**
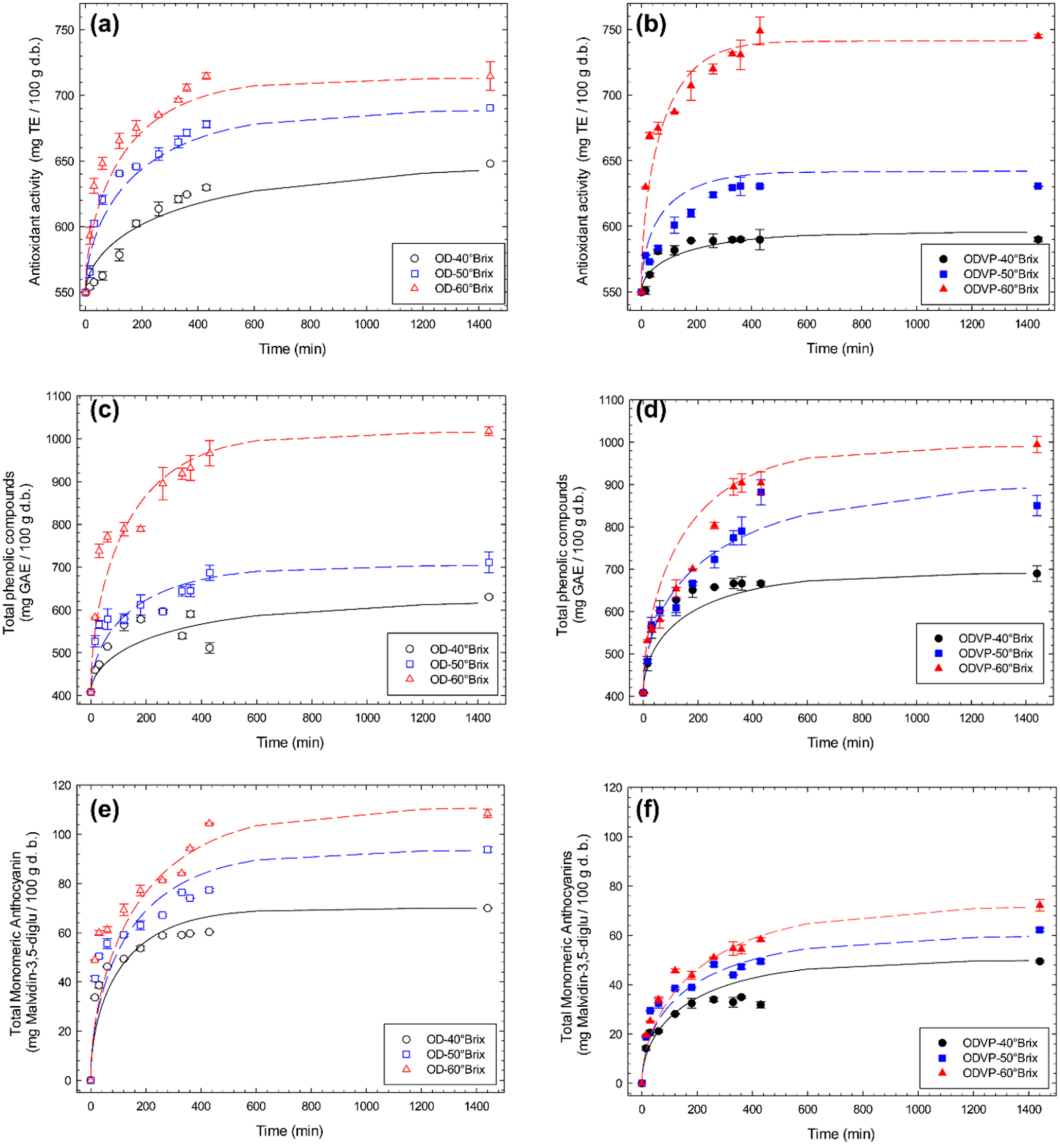
Evolution of bioactive compounds transfer of: antioxidant activity during (a) OD and (b) ODVP, of total phenolic compounds during (c) OD and (d) ODVP and of total monomeric anthocyanins during (e) OD and (f) ODVP of apple with grape juice concentrate at 40 °C.

Similarly, it was found that during OD of green papaya in blackberry juice solutions added with different sucrose concentrations, the amount of solute increased with increasing sucrose concentration;^40^ however, the amount of total monomeric anthocyanins decreased because sucrose incorporation was favored, this is mainly because the molecular weight of sucrose (342.3 g/mol) is lower than that of some anthocyanins. Previous studies have reported higher incorporation of total monomeric anthocyanins during OD at atmospheric pressure than in vacuum pulse-assisted processes of apples with pomegranate cryo-concentrated juice.^41^

This could explain why the ODVP-processed samples in our study achieved higher total soluble solid values and lower total monomeric anthocyanin values than the OD-processed samples. To achieve a sweet product, low molecular weight sweeteners were used in the osmotic solutions. For a product rich in bioactive compounds, limit the use of these sweeteners.^40^

On the other hand, the incorporation rates of AA, TPC, and TMA of the samples processed with ODVP showed a reduction with respect to OD; González-Pérez et al.^20^ associated this behavior to the accumulation of solutes on the external surface of the samples, which reduce the incorporation of bioactive compounds and favor the incorporation of total soluble solids. In the case of osmotically dehydrated samples at high concentrations, this behavior is associated with the viscosity of the osmotic agent; since it is very viscous, incorporation into the pores of the samples is almost nil. Pasławska et al.^2^ reported a reduction of antioxidant activity values and total phenolic compounds during ODVP of apple with apple-pear juice. They attributed this reduction to the leaching of bioactive compounds in the water outflow during the decompression stage. The authors found that using osmotic solutions with bioactive compounds (13.2 °Brix apple-pear juice) compensated for the bioactive compounds leached during the decompression process.

### 3.3 Effective Diffusion

Effective diffusion estimated with model solution considering shrinkage during process allowed us to describe the WL and SG, of bioactive compounds with correlation coefficients, *R* ^2^ > 0.80 (Table 2). The effective water diffusivity values for OD and ODVP-processed samples were 1.6–2.06 × 10^–10^ m^2^/s (*R* ^2^ > 0.872) and 1.8–2.12 × 10^–10^ m^2^/s (*R* ^2^ > 0.890), respectively. Water diffusivity values increased (*p* ≤ 0.05) with increasing osmotic agent concentration due to the osmotic pressure caused by a high difference between the concentrations of fresh food matrix and the osmotic solution.^5^ Effective water diffusivity of samples processed with ODVP was higher (*p* ≤ 0.05) than those processed at OD at the same osmotic agent concentration (Table 2) similar to that reported for guavas var. Red in 60 °Brix sucrose (OD and ODVP with 100 mmHg of vacuum pressure),^27^ and apple slices var. Granny Smith using 9 g of sucrose and 1 g of emulsion water in oil emulsion (9.98 × 10^8^ CFU of *Lactobacillus rhamnosus* LC705 and 62 g of grape seed oil/mL). Corrêa et al.^27^ associated this behavior to the decompression stage which allowed the removal of surface water from the sample resulting in an increase in water diffusivity and a reduction in solute diffusivity.

**Table 2:** Diffusivity (D) Parameter during Osmotic Procedures^a^

	Osmodehydration			Osmodehydration assisted with vacuum		
Diffusivity	40 °Brix	50 °Brix	60 °Brix	40 °Brix	50 °Brix	60 °Brix
**DW × 10**^**10**^ (m^2^/s)	1.66 ± 0.02cB	1.84 ± 0.02bF	2.06 ± 0.01aJ	1.80 ± 0.04hA	1.95 ± 0.02gE	2.12 ± 0.03fI
*R* ^2^	0.915	0.891	0.872	0.900	0.905	0.890
RMSE	0.240	0.291	0.330	0.244	0.244	0.263
*e*(*D*)	303.31	294.24	284.08	350.67	333.08	314.48
**DS × 10**^**10**^ (m^2^/s)	2.18 ± 0.02aA	2.03 ± 0.02bE	1.69 ± 0.03cI	1.54 ± 0.03gB	1.55 ± 0.02gF	1.66 ± 0.08fI
*R* ^2^	0.926	0.928	0.937	0.989	0.957	0.949
RMSE	0.236	0.211	0.181	0.028	0.124	0.152
*e*(*D*)	48.39	134.19	255.68	155.71	225.87	221.45
**DAA × 10**^**10**^ (m^2^/s)	0.84 ± 0.02cB	1.58 ± 0.02bF	2.10 ± 0.03aJ	2.32 ± 0.03hA	4.23 ± 0.01gE	4.39 ± 0.01fI
*R* ^2^	0.992	0.915	0.826	0.900	0.962	0.830
RMSE	0.015	0.199	0.292	0.179	0.105	0.421
*e*(*D*)	388.93	298.80	300.57	245.73	134.56	165.08
**DTPC × 10**^**10**^ (m^2^/s)	1.06 ± 0.02cB	2.00 ± 0.00bE	2.17 ± 0.03aI	2.12 ± 0.02fA	1.23 ± 0.00hF	1.87 ± 0.02gJ
*R* ^2^	0.912	0.836	0.800	0.910	0.944	0.982
RMSE	0.170	0.335	0.399	0.390	0.051	0.027
*e*(*D*)	1274.53	301.35	237.79	174.43	347.8	224.55
**DTMA × 10**^**10**^ (m^2^/s)	3.06 ± 0.01aA	2.08 ± 0.05bE	1.59 ± 0.04cI	2.02 ± 0.02fB	1.72 ± 0.02gF	1.30 ± 0.02hJ
*R* ^2^	0.852	0.800	0.801	0.852	0.801	0.870
RMSE	0.216	0.422	0.373	0.370	0.370	0.230
*e*(*D*)	312.97	384.09	388.18	444.16	478.78	429.08

a DA, DS, DAA, DTPC, and DTMA are water, solutes, antioxidant activity, total phenolic compounds, and total monomeric anthocyanins diffusivity, respectively. In the same row, lowercase letters (a, b, c, or f, g, h) indicate a significant difference (*p* ≤ 0.05) when comparing osmotic solution concentration at the same treatment. Additionally, capital letters (A, B or E, F or I, J) indicate significant differences (*p* ≤ 0.05) when comparing the type of treatment at the same osmotic solution concentration. *e*(*D*) is the ratio between diffusion without (Table S1) and with consideration of the shrinkage of the product during the process.

The effective solute diffusivity values of samples processed using ODVP (1.54–1.66 × 10^–10^ m^2^/s, *R* ^2^ > 0.949) were lower (*p* ≤ 0.05) than those of samples processed with OD (1.69–2.18 × 10^–10^ m^2^/s, *R* ^2^ > 0.926) (Table 2). This indicated that the difference between the osmotic pressure of the food matrix and the osmotic solution was lower than that of the samples osmodehydrated at atmospheric pressure as found during the 30 min process of OD and ODVP (pressure reduction of 100 mmHg) of apple var. Granny Smith in extracts of *Hibiscus sabdariffa* calyxes-water.^42^ Md Salim et al.^43^ reported that the greater the difference between the osmotic pressure of the food matrix and the osmotic solution, the higher the solute diffusivity value. On the other hand, no significant differences (*p* > 0.05) were found between the effective solute diffusivity of samples processed at ODVP and OD using 60° solutions, González-Pérez et al.^20^ associate this behavior to the fact that the absolute pressure of the system is not low enough to incorporate viscous osmotic solutions.

The DTMA was higher in OD processes (1.59–3.06 × 10^–10^ m^2^/s, *R* ^2^ > 0.80) than ODVP (1.30–2.02 × 10^–10^ m^2^/s, *R* ^2^ > 0.801) and decreased with increasing osmotic agent concentration. Also, the highest DTMA was reached in ODVP procedures with 40 °Brix, and it is attributed to the uniform impregnation described with the EC curve (Figure 4). In the other experimental conditions, the solute accumulation on the external part of product reduced the DTMA. This behavior was similar Indian gooseberry (*Emblica officinalis*) impregnated with 5.5, 10, and 20 °Brix of sucrose-anthocyanin extracts solutions (168.63 mg/L cyanidin-3-glucoside from kokumi).^44^

A decrease in the diffusivities of total phenolic compounds was observed for samples processed with ODVP (1.23–2.12 × 10^–10^ m^2^/s, *R* ^2^ > 0.91) due to the increased concentration of the osmotic solutions. The DTPC of the samples processed with ODVP and solutions of 50 and 60 °Brix were lower than those determined for the samples processed with OD at the same concentration, this may be because the vacuum treatment increased the incorporation of solutes on the surface of the product which caused a reduction of solute incorporation.^9^ Wang et al.^45^ found a reduction in the rate of solute transfer during ODVP processes due to the increase in the concentration of the osmotic solution, the authors attributed this reduction to the viscosity of the solution, the more viscous the solution, the longer it takes to incorporate the osmotic solution into the pores of the food matrix during the decompression stage.

In both treatments, an increase in antioxidant activity diffusivity was observed due to increasing concentration. The use of ODVP (2.32–4.37 × 10^–10^ m^2^/s, *R* ^2^ > 0.830) showed higher antioxidant activity impregnation diffusivity values than those estimated for OD (0.84–2.10 × 10^–10^ m^2^/s, *R* ^2^ > 0.826), similar to that reported for apple with apple juice impregnation (30–60 mmHg).^2^

Cranck’s analytical solution model for nonshrinkage solids of cubic geometry caused an overestimation of diffusion coefficients under all the tested similarity criteria. Effective diffusion estimated with nonshrinkage products reached *R* ^2^ > 0.473 (Table S1), which implies that it is necessary to consider the product size to increase the fitness of mass transfer coefficients. The study compared the ratio between diffusion and diffusion without consideration (Table S1) and consideration of product shrinkage during the process to assess the impact of neglecting shrinkage on the estimation of diffusivity (Table 2). The findings revealed values ranging from 48.39 to 478 (Table 2), indicating an overestimation of this parameter. By adjusting for the actual size, the diffusivity values of the various components analyzed were reduced, resulting in a more accurate. González-Pérez et al.^46^ found that not considering shrinkage during the osmodehydration process of white mushroom pileus with 25% sodium chloride solutions caused an overestimation of 31.8 to 113, and when considering shrinkage in the diffusivity estimation, the correlation coefficients increased from *R* ^2^ > 0.81 to *R* ^2^ > 0.85. Similarly, Zecchi and Gerla^47^ found that diffusion coefficients determined considering real shape and shrinkage during the osmodehydration process of tomato halves in sucrose-sodium chloride solutions are lower than values considering a nonshrinking solid. In addition to shrinkage, the apple cubes also exhibited deformation. The effect of this deformation is beyond the scope of this work. Therefore, future research should consider these morphological changes when determining the diffusivity coefficients to more accurately approximate the actual values.

On the other hand, a strong statistical correlation was identified between the diffusivity of different components and the Max-Min ECC values for treatment performed with 40 °Brix grape juice; a significant correlation (*R* ^2^ > 0.80) was found between diffusivity and ECC Max-Min values (Figure 8). To validate this, a Pearson's correlation analysis was conducted. Table 3 shows Pearson's correlation coefficients between the Euler Characteristic Max-Min and the diffusivities of water (DW), solutes (DS), antioxidant activity (DAA), total phenolic compounds (DTPC), and total monomeric anthocyanins (DTMA) during OD, both with and without vacuum assistance, at 40, 50, and 60 °Brix. The results reveal robust associations between ECC and the diffusivities of the studied elements. Most correlations are strongly positive, indicating a direct relationship. For example, water diffusivity (DW) correlates highly with the Euler characteristic, with values such as 0.9573 (40 °Brix, OD) and 0.9872 (40 °Brix, vacuum-assisted). Similarly, diffusivities of total phenolic compounds (DTPC) and total monomeric anthocyanins (DTMA) show strong positive correlations, reaching 0.9695 (50 °Brix, OD) and 0.9902 (60 °Brix, OD), respectively. However, at 60 °Brix under vacuum-assisted conditions, significant negative correlations are observed, such as −0.9904 (DW), −0.995 (DS), and −0.9795 (DAA), suggesting an inverse relationship in this specific scenario.

**Figure 8:**
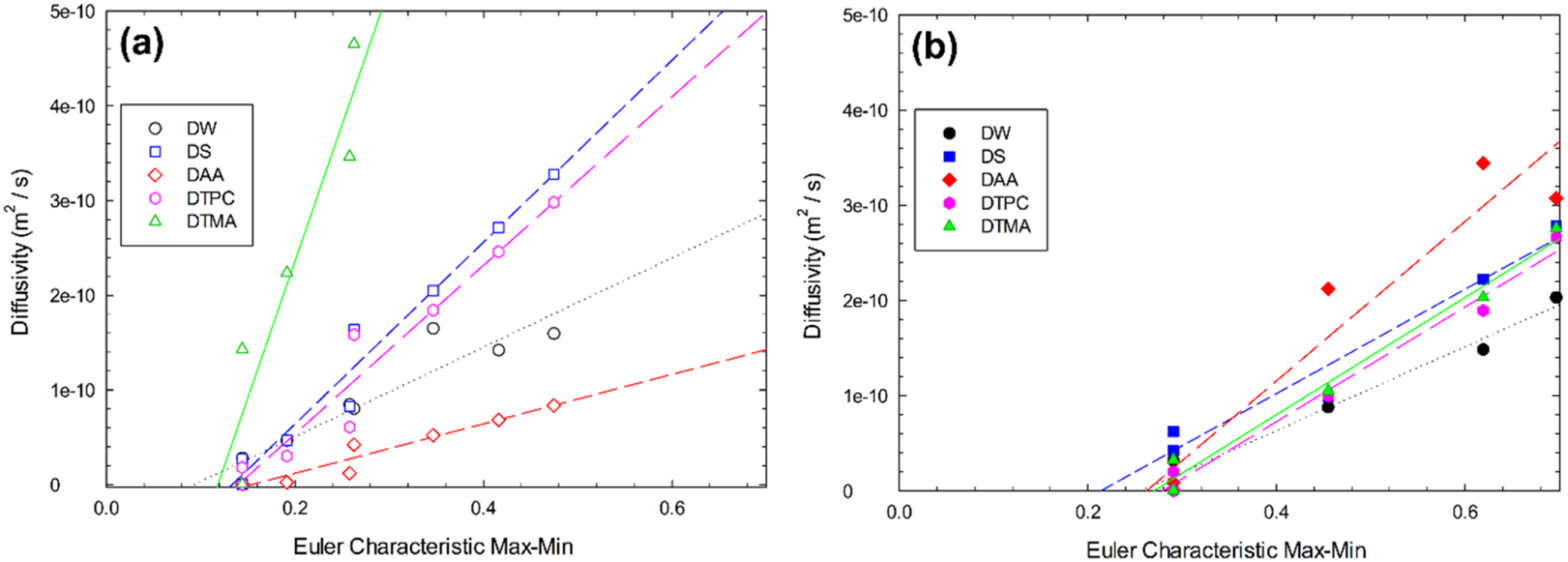
Euler characteristic Max-Min *against* diffusivity during osmotic procedures using 40 °Brix of grape juice (a) OD and (b) ODVP.

**Table 3:** Pearson's Correlation Euler Characteristic Max-Min and Diffusivity^a^

	Euler Characteristic Max-Min*					
	Osmodehydration			Osmodehydration assisted with vacuum		
Diffusivity	40 °Brix	50 °Brix	60 °Brix	40 °Brix	50 °Brix	60 °Brix
**DW**	0.9573	0.9215	0.8825	0.9872	0.8705	–0.9904
**DS**	0.8880	0.9518	0.9077	0.9802	0.7508	–0.995
**DAA**	0.7858	0.9592	0.9331	0.9586	0.7523	–0.9795
**DTPC**	0.8312	0.9695	0.8536	0.9929	0.8841	–0.9567
**DTMA**	0.9365	0.9654	0.9902	0.9912	0.8121	–0.9765

a DW, DS, DAA, DTPC, and DTMA are water, solutes, antioxidant activity, total phenolic compounds, and total monomeric anthocyanins diffusivity, respectively.

This supports the hypothesis that highly viscous osmotic agents result in nonuniform impregnation, characterized by a high concentration of solutes on the exterior, which impedes solute incorporation or water elimination. Additionally, solutes are redistributed due to concentration differences between the exterior and the center of the product, thereby reducing the solute concentration on the exterior and facilitating further solute incorporation. Previous studies have reported nonuniform impregnation during vacuum impregnation of apples with viscous osmotic agents such as 2% methylene violet with starch syrup (400 mPas)^9^ and 40–60 °Brix grape juice (18.31–64.31 mPas).^20^ In both instances, accumulation was observed on the exterior, which was linked to a reduced extent of solute incorporation. The ECC results indicate that continuing the OD process post-impregnation allows the redistribution of impregnated solutes, thereby enhancing solute incorporation. The strong correlation between the ECC Max-Min values and diffusivity coefficients is of relevance, as this indicates that real-time images coupled to TDA could be used as a method for enabling the nonintrusive monitoring of impregnation experiments.

## 4 Conclusions

Topological image analysis was used for analyzing the diffusive behavior during the impregnation of juice in apples. The overall findings suggest that using a topological descriptor known as the Euler characteristic curve (ECC) provides a powerful and nonintrusive alternative for real-time monitoring and analysis of impregnation experiments. The ECC indicates that ODVP experiments led to more abrupt and nonuniform impregnation than atmospheric pressure. OD-40 experiments exhibited ECC with less pronounced peaks, indicating a uniform solute impregnation. Conversely, experiments with nonuniform impregnations demonstrated lower effective SG and TMA diffusivity coefficients than those with uniform impregnation. Applying these topological tools may significantly contribute to predicting whether an accumulation of solutes reduces the transfer rate of certain solutes. The performed analysis indicates that in nonuniform impregnation with high viscosity, as in ODVP60 experiments, solute accumulation on the external part of the product reduces the effective diffusion of diffusive substances and redistribution of solutes increases diffusivity. In future research, it is recommended to analyze the ECC of products osmodehydrated with viscous liquids to determine the time required to find no significant differences in these curves and proceed to a more efficient drying process. Finally, it is recommended to incorporate the deformation of samples during osmotic procedures into the calculation of effective diffusivity to obtain estimates of values closer to reality.

## Supplementary Material



## Data Availability

All data generated during this study have been included in the manuscript and the Supporting Information.

## Notes

Temporal evolution of the images in original color, evolution of topology for different processing conditions, and diffusivity (*D*) parameter during osmotic procedures without considering shrinkage (PDF)

## Nomenclature Variables

AA: Antioxidant activity (mg TE/100 g d.b.)
AA_0_: Initial antioxidant activity (550 ± 0.24 mg TE/100 g d.b)
AA–AA_0_: Increase in antioxidant activity (mg TE/100 g d.b)
Abs_DPPH_: Absorbance of DPPH solution
Abs_sample_: Absorbance of sample
*a*_*w*_: Water activity (dimensionless)
Brix: Total soluble solids content (g soluble solids/100 g solution)
C: Concentration of the osmotic solution (°Brix)
D: Effective diffusivity (m^2^/s)
*d.b.*: dry basis
DAA: Antioxidant activity diffusivity (m^2^/s)
DS: Solute diffusivity (m^2^/s)
DTMA: Total monomeric anthocyanins diffusivity (m^2^/s)
DTPC: Total phenolic compounds diffusivity (m^2^/s)
DW: Water diffusivity (m^2^/s)
EC: Euler Characteristic
ECC Max-Min: Difference between maximum and minimum peaks in the Euler characteristic curve
ECC: Euler characteristic curve
*e*(*D*): ratio between diffusion without and with consider shrinkage of product during the process
GAE: Gallic Acid Equivalents
GJC: Grape juice concentrate
i: number of terms
*k*_1_: Peleg’s model reciprocal of rate constant in s/(g water/g fresh product) for WL, s/(g solutes/g fresh product) for SG, s/(mg TE/100 g product *d.b*.) for AA, s/(mg GAE/100 g product *d.b*.) for TPC or s/(mg Mlv/100 g product *d.b*.) for TMA
*k*_2_: Peleg’s model constant of capacity in 1/(g water/g fresh product) for WL, 1/(g solutes/g fresh product) for SG, 1/(mg TE/100 g product *d.b*.) for AA, 1/(mg GAE/100 g product *d.b*.) for TPC or 1/(mg Mlv/100 g product *d.b*.) for TMA
*L, L*_1_, *L*_2_, *L*_3_: Half of sample length (m)
*L*_0_, *L*_1,0_, *L*_2,0_, *L*_3,0_: Half of initial sample length (m)
*m*: Mass (g)
*M*: Moisture content (g water/g sample)
*M*_0_: Initial moisture content of the fresh sample (g water/g sample)
Mlv: Malvidin-3,5-diglucoside
*m*_OP_: Mass of osmotically processed sample (g)
*M*_OP_: Moisture content of osmotically processed sample (g water/g sample)
*m*_p0_: Initial mass of the fresh sample (g)
OD: Osmotic dehydration
ODVP: Osmotic dehydration assisted with vacuum pulses
*P*_abs_: Absolute pressure (mmHg)
PVOD: Pulsed Vacuum Osmotic Dehydration
*R* ^2^: Coefficient of determination (dimensionless)
*r* ^2^: Squared value of the Pearson correlation coefficient
RMSE: Root Mean Square Error
SG: Solute gain (g solutes/g fresh product)
SG–SG_0_: Increase in solutes gain (g solutes/g fresh product)
*t*: Time (s)
TDA: Topological Data Analysis
TE: Trolox Equivalents
TMA: Total monomeric anthocyanins (mg Malvidin-3,5-Diglucoside/100 g d.b.)
TMA_0_: Initial total monomeric anthocyanins (mg mLv/100 g d.b.)
TMA–TMA_0_: Increase in total monomeric anthocyanins (mg mLv/100 g d.b.)
TPC: Total phenolic compounds (mg GAE/100 g d.b.)
TPC_0_: Initial total phenolic compounds (415.97 ± 0.99 mg GAE/100 g d.b.)
TPC–TPC_0_: Increase in total phenolic compounds (mg GAE/100 g d.b.)
V: Volume at time t (m^3^)
v/v: volume/volume
V/V_0_: Dimensionless volume (dimensionless)
V_0_: Initial volume (m^3^)
w.b.: wet basis
w/w: weight/weight
WL: Water loss (g water/g fresh product)
WL–WL_0_: Increase in water loss (g water/g fresh product)
*Y*_e_: Increase in mass transfer parameter at equilibrium
*Y*_e_: Equilibrium condition of the increasing of different mass transfer parameters (mass transfer units)
*Y*_e_*: Mass transfer parameter at equilibrium (g water/g fresh product for WL; g solutes/g fresh product for SG; mg TE/100 g d.b. for *AA*; mg GAE/100 g d.b. for *TPC*; mg Mlv/100 g d.b. for TMA)
*Y*_e_ = *Y*_e_*–*Y*_0_: This describes the equilibrium condition (g water/g fresh product for WL; g solutes/g fresh product for SG; mg TE/100 g d.b. for *AA*; mg GAE/100 g d.b. for *TPC*; mg Mlv/100 g d.b. for TMA)
*Y_j_*: Mass transfer parameter at a given time (g water/g fresh product for WL; g solutes/g fresh product for SG; mg TE/100 g d.b. for *AA*; mg GAE/100 g d.b. for *TPC*; mg Mlv/100 g d.b. for TMA)
*Y*_*j*,0_: Initial condition of the increasing of different mass transfer parameters (g water/g fresh product for WL; g solutes/g fresh product for SG; mg TE/100 g d.b. for *AA*; mg GAE/100 g d.b. for TPC; mg Mlv/100 g d.b. for TMA)
*Y*_*j*,OP_: Mass change of the diffusing substance j per mass of fresh sample in OD or ODVP (g water/g fresh product for WL; g solutes/g fresh product for SG; mg TE/100 g d.b. for AA; mg GAE/100 g d.b. for TPC; mg Mlv/100 g d.b. for TMA)
*Y_j_*–*Y*_j0_: Increase in different mass transfer parameters (g water/g fresh product for WL; g solutes/g fresh product for SG; mg TE/100 g d.b. for AA; mg GAE/100 g d.b. for TPC; mg Mlv/100 g d.b. for TMA)

## Greek symbols

τ*_j_*: Fourier mass transfer number (dimensionless) for substance j
τ*_j_* *: Comprehensive Fourier mass transfer number (dimensionless) for substance j
Ψ*_j_*: Dimensionless moisture ratio (dimensionless) for substance j

## Subscripts

0: at the beginning of the osmotic process
e: at equilibrium
j: of substance (water, total soluble solids, solutes AA, TPC, or TMA)
OP: in osmotic procedures (OD or ODVP)
