# Supplementary material for: Implementing Topological Data Analysis for Monitoring Mass Transfer during Vacuum-Assisted Osmotic Dehydration of Apples
Source: ACS Omega. 2025 Jul 8;10(28):30137–54. doi: 10.1021/acsomega.5c00531 (PMC12290977; doi:10.1021/acsomega.5c00531)
Supplement: Supplementary file 1 [file ao5c00531_si_001.pdf]

Supplementary data to:

Implementing Topological Data Analysis for monitoring Mass Transfer during Vacuum-Assisted Osmotic Dehydration of Apples

Julio. E. González-Pérez [a,b], Shengli Jiang [c], Oscar Jiménez-González [a,d], Víctor M Zavala [c], Aarón Romo-Hernández [a], José Á. Guerrero-Beltrán [a], Aurelio López-Malo [a], & Nelly Ramírez-Corona [a*]

[a] Department of Chemical, Food and Environmental Engineering, Universidad de las Américas Puebla, Ex hacienda de Santa Catarina Mártir, San Andrés Cholula, Puebla, Mexico 72810. [b]Tecnologico de Monterrey, School of Engineering and Sciences, Ave. Eugenio Garza Sada 2501 Sur, Col: Tecnológico, Monterrey, N.L., Mexico, 6470. [c]Department of Chemical and Biological Engineering, University of Wisconsin-Madison, Madison, WI, 53706, United States. [d] Faculty of Gastronomy, Universidad Popular Autónoma del Estado de Puebla, 21 Sur No. 1103, Barrio de Santiago, Puebla 72410, Mexico

* Corresponding author: nelly.ramirez@udlap.mx

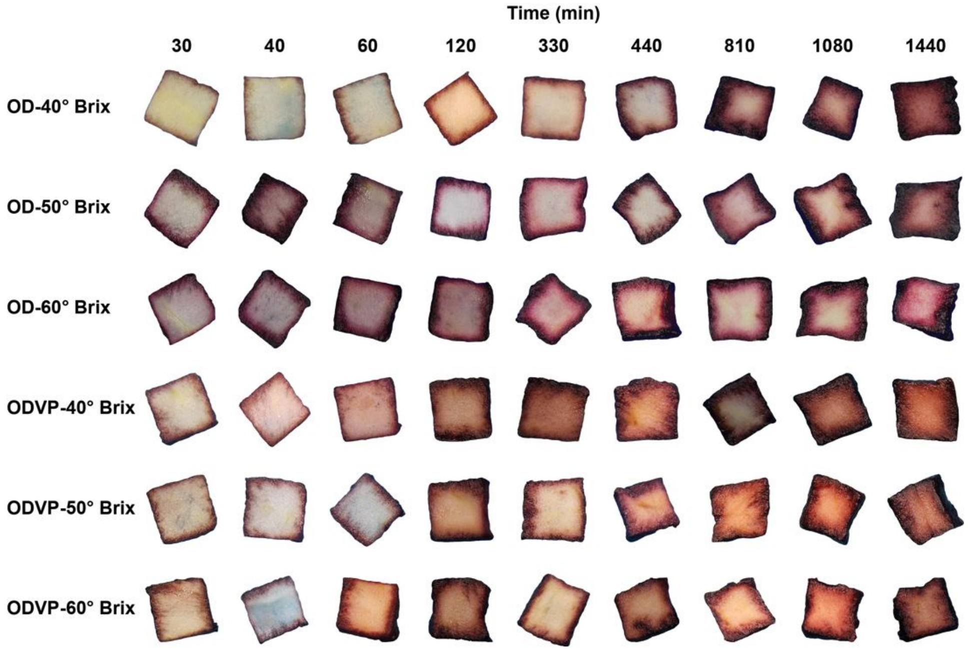

Figure S1. Temporal evolution of image samples under different processing conditions in original color.

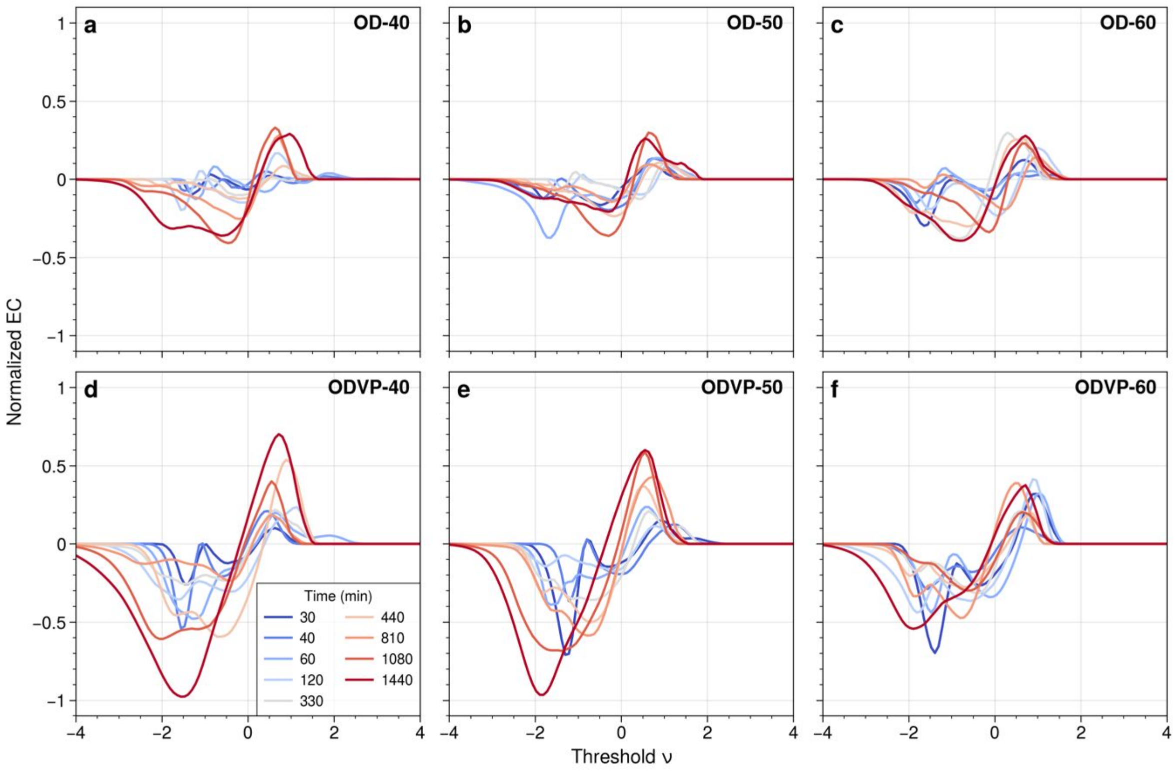

Figure S2. Temporal evolution of topology for different processing conditions. **(a)** osmodehydrated with 40°Brix (OD-40), **(b)** osmodehydrated with 50°Brix (OD-50), **(c)** osmodehydrated with 60°Brix (OD-60)), **(d)** osmodehydrated assisted with vacuum pulses using 40°Brix (ODVP-40), **(e)** osmodehydrated assisted with vacuum pulses using 50°Brix (ODVP-50), and **(f)** osmodehydrated assisted with vacuum pulses using 60°Brix (ODVP-60).

Table S1. Diffusivity (D) parameter during osmotic procedures without considering shrinkage.

Diffusivity	Osmodehydration			Osmodehydration assisted with vaccum		
	40 °Brix	50 °Brix	60 °Brix	40 °Brix	50 °Brix	60 °Brix
**DW ×10^10** (m^2 / s)	503.5±8.57cB	541.4±4.95bF	585.2±7.17aJ	631.2±6.26hA	649.5±6.16gE	666.7±597.8fI
R^2	0.903	0.598	0.772	0.8134	0.901	0.81
RMSE	0.877	0.302	0.802	0.654	0.593	0.602
**DS×10^10** (m^2 / s)	105.5±1.73cB	272.4±5.14bF	432.1±2.95aI	239.8±4.71hA	350.1±3.88gE	367.6±2.26fJ
R^2	0.723	0.642	0.909	0.680429	0.845	0.906
RMSE	0.849	0.798	0.338	0.821	0.424	0.217
**DAA×10^10** (m^2 / s)	326.7±1.51cB	472.1±0.54bF	631.2±0.97aJ	570.1±5.64gA	569.2±5.25gE	724.7±1.56fI
R^2	0.892	0.903	0.802	0.9	0.9	0.803
RMSE	0.177	0.63	0.98	0.66	0.51	0.125
**DTPC ×10^10** (m^2 / s)	1351±2.21aA	602.7±1.331bE	516±1.42cI	369.8±9.97gB	427.8±9.52fF	419.9±4.48fJ
R^2	0.849	0.73	0.6756	0.6397	0.825	0.951
RMSE	0.19	0.986	0.892	0.492	0.111	0.043
**DTMA ×10^10** (m^2 / s)	957.7±2.67aA	798.9±1.51bE	617.2±1.97cI	897.2±2.02fB	823.5±1.13gF	557.8±9.64hJ
R^2	0.736	0.832	0.473	0.792	0.797	0.851
RMSE	0.588	0.795	0.158	0.36	0.364	0.94

DA, DS, DAA, DTPC, DTMA are water, solutes, antioxidant activity, total phenolic compounds, and total monomeric anthocyanins diffusivity, respectively. In the same row, lowercase letters (a, b, c, or f, g, h) indicate a significant difference ($p \leq 0.05$) when comparing osmotic solution concentration at the same treatment. Additionally, capital letters (A, B or E, F or I, J) indicate significant differences ($p \leq 0.05$) when comparing the type of treatment at the same osmotic solution concentration.
